# Development of Interdisciplinary SynZyme CURE: A Bridge between Organic Synthesis, Protein Biochemistry, and Drug Discovery

**DOI:** 10.1021/acs.jchemed.6c00350

**Published:** 2026-08-20

**Authors:** Scott Westervelt, Grace M. Bennett, Mark V. C. dela Cerna, Shainaz Landge

**Affiliations:** † Department of Biochemistry, Chemistry, and Physics, 7604 Georgia Southern University, 521 College of Education Drive, Statesboro, Georgia 30458, United States; ‡ Department of Biochemistry, Chemistry, and Physics, Georgia Southern University, 11935 Abercorn Street, Savannah, Georgia 31419, United States

**Keywords:** Upper-Division Undergraduate, Interdisciplinary/Multidisciplinary, Organic Chemistry, Biochemistry, Inquiry-Based/Discovery Learning, Problem Solving/Decision Making, Synthesis, CURE, Bioinstrumental, Enzyme Inhibition

## Abstract

The SynZyme Course-Based Undergraduate Research Experience (CURE) was developed to immerse students in an interdisciplinary semester-long project integrating organic synthesis and enzyme biochemistry within the context of early stage drug development. Embedded within a bioinstrumental chemistry course, this CURE also emphasizes the practical use of instrumentation in chemical and biochemical research. Eight undergraduate students synthesized a series of five novel triazole-based compounds and performed detailed molecular characterization to confirm purity and identity. Students then performed ADMET analysis and molecular docking before evaluating the inhibitory activity of the compounds against two target bacterial protein tyrosine phosphatases. Student Assessment of their Learning Gains (SALG), along with the Undergraduate Research Student Self-Assessment (URSSA) instrument, was used to evaluate the gains and experiences of the participants. Data revealed increased confidence in conducting independent research and contributing meaningfully to a research project. It also highlights the potential to promote a deeper understanding, skill development, and research competency among undergraduate students. Overall, the SynZyme CURE demonstrates the effectiveness of course-based research in fostering scientific identity, strengthening interdisciplinary literacy, and preparing undergraduates for real-world research environments.

## Graphical abstract

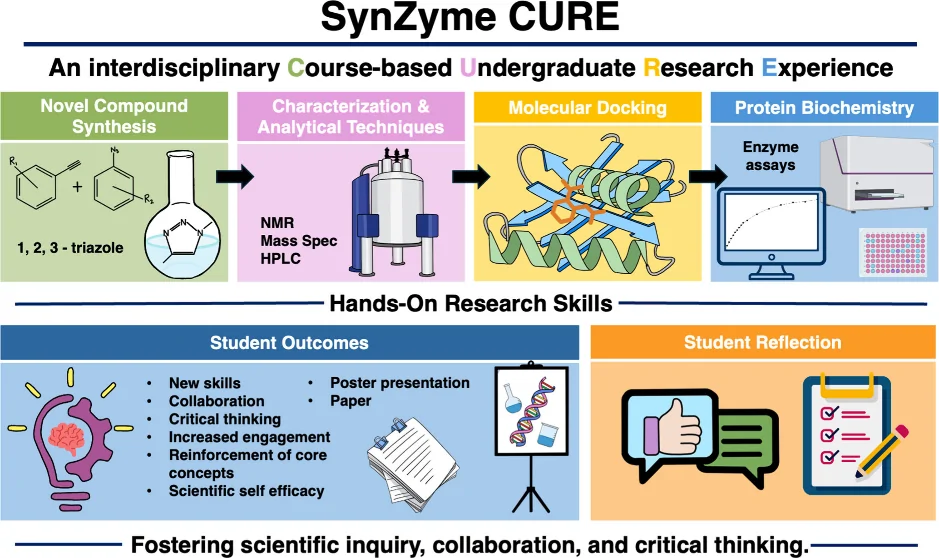

## Introduction

Practical training in STEM education is typically embedded in programs of study through laboratory courses. However, there have been criticisms, particularly on “cookbook” laboratories, and how outcomes align with practical learning objectives.^1,2^ Another route for students to enrich practical knowledge is engaging in faculty-led research. Unfortunately, there is a problem of scalability involving the number of opportunities available. Course-based undergraduate research experiences (CUREs), which incorporate authentic research into the undergraduate curriculum, address these concerns.^2–5^

Declining engagement is a challenge that plagues higher education, including in laboratory-intensive curricula where students struggle to connect concepts to practice and real-world applications.^6–8^ CUREs directly address these by satisfying the central aims of Scholarship of Teaching and Learning (SoTL): designing learning environments that move students beyond compliance and toward genuine investment.^9^ These enable students to appreciate what they learn, why it matters, and how knowledge is generated and applied. CUREs immerse students in experiences that mirror what happens in a research lab from developing questions, generating data, troubleshooting, analyzing, and communicating findings. They improve not only conceptual understanding but also gain intangible skills, such as communication and teamwork. Importantly, it can help students articulate competencies.^10–13^ CUREs enable participation in an independent and authentic discovery process. They alleviate challenges to participating in research, including the opportunity cost of choosing research over a job and lack of awareness of opportunities.

Chemistry CUREs have expanded rapidly with models implemented involving organic synthesis, analytical chemistry, chemical biology, biochemistry, materials science, and computational chemistry.^14^ A review from 2016 to 2022 reports on the implementation of some of these.^15^ A natural evolution, addressing demands for interdisciplinarity, includes bridging CUREs with an overarching theme supporting long-term research where students are exposed to multidisciplinary approaches providing mastery in more than one area. Examples include CUREs linking environmental toxicology and analytical chemistry, microbiology and biochemistry, and biocatalysis and organic chemistry.^16–20^ The need for online delivery due to COVID-19 also catalyzed the development of hybrid and remote CUREs.^20–22^ Continued development of interdisciplinary CUREs is necessary, as the modern workforce demands a multitude of skills that may not be sufficiently addressed by traditional laboratory courses.

### Course and SynZyme Background

This CURE was implemented as the laboratory section for upper-division instrumental analysis offered to chemistry/biochemistry seniors at a Research (R2) Primarily Undergraduate Institution (PUI). The course historically implemented ‘cookbook’ hands-on exercises. This CURE will provide students with practical instrumentation experience in the context of a cohesive, semester-long project. As the course is taught to biochemistry and chemistry majors, it bridges applications relevant to both programs. Decline in enrollment necessitated cross-listing the courses. This CURE makes the most out of the situation by providing interdisciplinary training to students in both majors. Thus, SynZyme was born!

SynZyme bridges organic synthesis and protein biochemistry, led by two faculty pursuing research in these areas. The first module focuses on the synthesis of novel triazole-based compounds through click chemistry, microwave synthesis, and compound characterization. The second module focuses on purification of proteins, optimization of assays, and inhibition and binding assays. There is a focus on mastery of practical aspects of instrumentation, data analysis, and troubleshooting, as part of instrumental analysis. Students use several instrumentation including UV–vis and fluorescence spectroscopy, nuclear magnetic resonance (NMR) spectroscopy, liquid chromatography, microplate reader, and other research equipment, covered in the lecture (see Supporting Information - *Course Syllabus*). Students were introduced to docking and computational absorption, distribution, metabolism, excretion, and toxicology (ADMET) analysis to augment their work. The CURE required a final report in the form of an ACS-style manuscript and poster presented in a college-level symposium (see Supporting Information - *Student Final Poster Presentation Examples*). Scientific communication workshops were held to help improve student ability to communicate their work to peers and other experts.

### Research and Implementation

SynZyme combines synthetic chemistry and protein biochemistry to develop protein-targeted antimicrobials (see Supporting Information - *Schedule*). Faculty leads combined their expertise to provide an authentic interdisciplinary research experience. Participants were introduced to the global concern of antibiotic resistance and the importance of developing novel therapeutics to combat it.^23,24^ Using this as an anchor, the CURE contextualizes lab work within a therapeutic development framework that has relevance beyond the classroom. This is an important dimension of laboratory experiences within CUREs.^11^

Bacterial protein tyrosine phosphatases (PTP) are of emerging interest in antimicrobial development and were chosen as targets for SynZyme.^25^ Each pair was assigned one of two PTPs, both novel targets that have not been a subject of any drug discovery. While one has a crystal structure, the other has not been expressed recombinantly.^26^ Participants expressed the proteins in *E. coli* and purified them via affinity chromatography. Phosphatase activities of the proteins were evaluated using a synthetic substrate and vanadate as a general PTP inhibitor.^27^ On the chemistry side, participants synthesized 1,2,3-triazoles through one-step microwave-assisted click chemistry (Scheme 1).^28^ Students were briefly introduced to this approach and the works of Sharpless and Meldal (2022 Nobel Prize in Chemistry) to further exemplify broader impact of the work.^29^ Target triazoles are novel so success of synthesis is not guaranteed lending to potential higher impact and for the participants to be involved in publishable research. Molecules were characterized by melting point analysis, mass spectrometry, NMR, ultraviolet–visible and fluorescence spectroscopy, and X-ray crystallography. ADMET predictions and molecular docking were also performed, providing a bridge from molecules to therapeutic developments. Participants engaged in an authentic discovery process and were given safety training in accordance with department policies (see Supporting Information - *Sample Course Materials, Lab Safety*). While there are no unexpected and unusually high hazards, participants were required to wear PPE (gloves, lab coats, and goggles) in the lab. Participants engage in several research areas, primarily medicinal chemistry and biology/biochemistry, with principles from chemoinformatics and structural biology. Results from both modules are novel and will lead to *bona fide* drug discovery programs.

**Scheme 1:**
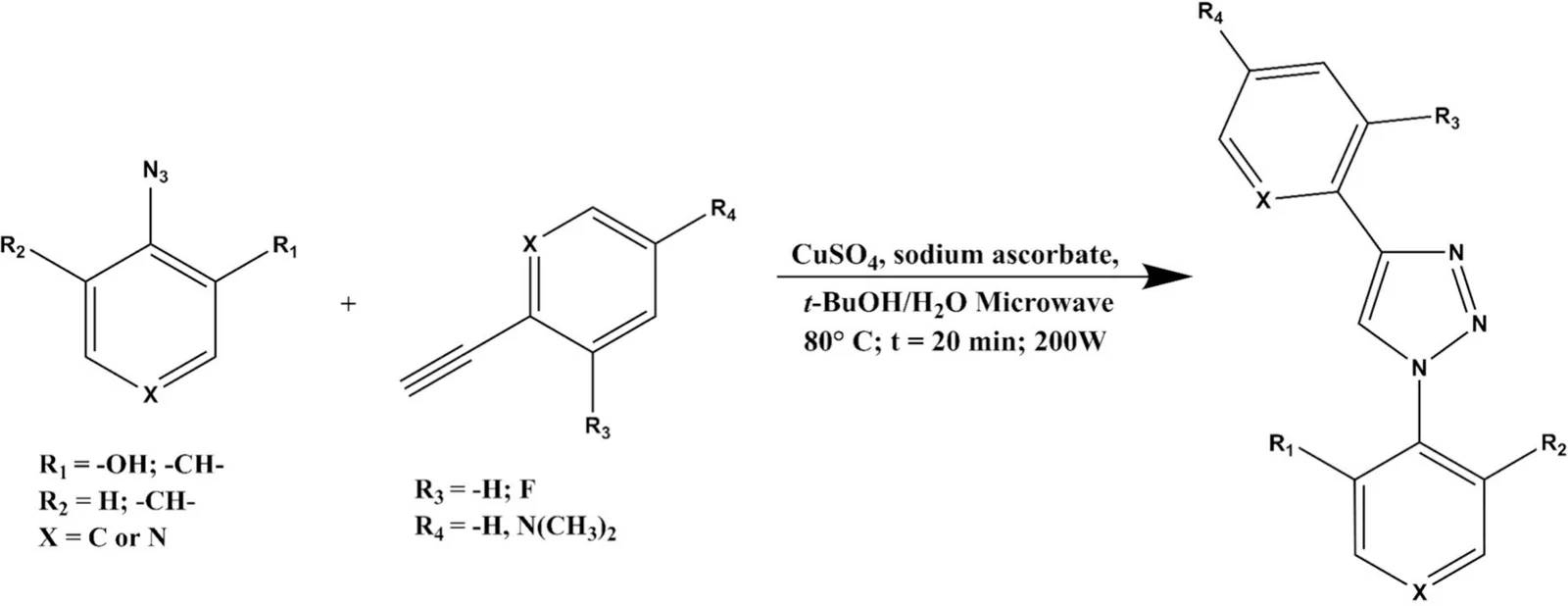
General Procedure for the Synthesis of 1,2,3-Triazoles

Instrumentation used throughout the CURE was covered in lecture. Students get significant practical time to characterize their proteins and compounds. The interdisciplinarity of the course enabled use of instrumentation in different contexts. For instance, liquid chromatography was used to purify proteins (FPLC) and characterize their molecules (LC/MS), and a spectrophotometer was used to quantify purified protein and evaluate fluorescence of the molecules. Students had substantial opportunities to use NMR to characterize small molecules and determine the crystal structure of their compounds. While protein NMR and crystallography were not accessible, practical experience provided a springboard for discussion in the lectures. This also embodies the iteration dimension of the CUREs.

Weekly meetings encouraged collaborative discussions, akin to research lab meetings, and participants had to take responsibility for and ownership of their work. They lead project design, planning, troubleshooting, and optimization, and data analysis with the instructors serving as guides.^11^ Knowing that several aspects of the project may not work the first time and without preprepared materials, flexible days were built into the semester for time to repeat experiments due to low yields, ambiguous results, or protocol optimization. Participants conduct research with real-life implications for combating antibiotic resistance. As there are no published discovery efforts against the targets and the molecules are novel, there is a high expectation for publications and presentations.

This paper describes outcomes from SynZyme, a model for interdisciplinary experiences for undergraduate research that can be replicated or adapted within a chemistry or biochemistry curriculum, integrating synthesis, enzyme inhibition, and instrumental analysis. We report learning gains and research-related outcomes that can be used to improve implementation. Data provide evidence that an integrated synthesis-to-assay course can foster engagement, scientific ownership, and scientific literacy while strengthening competency involving instrumentation and data analysis, aligning with SoTL priorities and addressing needs of modern chemical and biochemical education.

## Results and Discussion

### Participant Demographics and Institutional Review Board

The study consisted of eight students, evenly divided between men (50%) and women (50%). Half of the students identified as Asian (50%), with representation from Black or African American (25%) and White (37.5%). One was identified as Hispanic or Latino/Latina (12.5%). Two were juniors and six were seniors (see Supporting Information, Table 1). All authors had access to all deidentified data. All participants signed an informed consent document. As authorized in the Federal Policy for the Protection of Human Subjects, Institutional Review Board (IRB) reviewed and approved this study as exempt from the full review (*protocol* ***H26127***; see Supporting Information - *Informed Consent and Student Survey Recruitment Email*).

### Assessment Strategy for SynZyme CURE

We adapted the Undergraduate Research Student Self-Assessment (URSSA) and the Student Assessment of Learning Gains (SALG; Master Survey) instrument to evaluate student CURE experience (see Supporting Information - *Instrument/Survey*).

URSSA is designed to capture self-reported learning gains across cognitive, behavioral, and affective domains associated with undergraduate research experiences.^30^ Only items aligned with the objectives of the CURE were selected. Given a small cohort (*n* = 8), URSSA alone would not be enough to capture the overall experience. As such, SALG was also used to help understand student learning gains and motivations.^31^ This approach allowed us to assess student learning in areas such as research skills, personal growth, academic skill development, and attitudes toward scientific inquiry using a well-established framework tailored to the context of our course.

### Insights from the URSSA Instrument

Items aligning with the objectives of SynZyme were selected to evaluate the student’s learning experience (Figure 1). The gains scales items on the survey were rated on a five-point Likert scale ranging from 1 = no gain to 5 = a great gain covering gains in four domains: *Thinking and Working Like a Scientist*, *Personal Gains Related to Research, Skills*, and *Attitudes and Behaviors*. This has been used as a benchmark for student development in apprentice-style environments.^29^ We use data from a BIO-REU as a basis for comparison to understand whether outcomes from CUREs such as SynZyme mirror student-reported development from research.^32^

**Figure 1:**
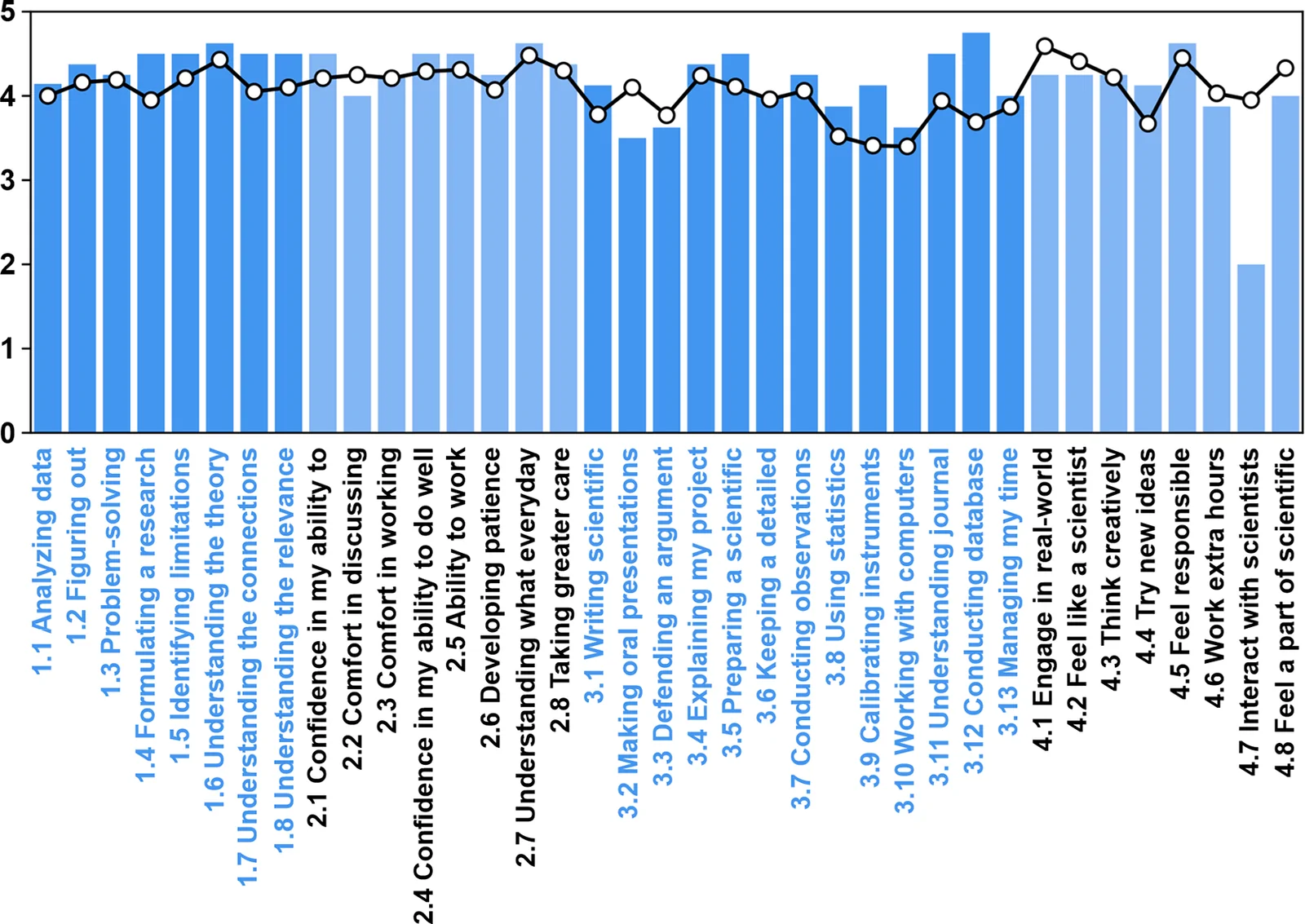
Student-Reported Gains from URSSA Domains. Results from the URSSA survey instrument from the current CURE­(bars, color difference only to highlight different domains). Current data are compared to results from Weston 2012 shown as a line plot.^30^ Complete set of survey questions are provided in the Supporting Information.

#### Domain 1: Thinking and Working Like a Scientist

Students perceived strong gains (mean of 4.42, all item means above 4.15) in this domain, which captures gains in understanding and applying the scientific process. Significant gains were observed in understanding of the theories and concepts guiding their projects, their ability to formulate research questions, identify project limitations, and recognize connections among different disciplines and coursework. Participants saw an abridged version of an authentic drug discovery pipeline, synthesized their molecules, and evaluated their ability to inhibit a target. The CURE was designed to encourage the ownership of their work. They selected (from a curated set) triazoles that they synthesized and also their target PTP. Participants were also given the opportunity to pursue questions beyond those required for the CURE. Some chose to synthesize additional compounds, perform in-depth characterization, or conduct computational investigations. The CURE evolved its own culture similar to that of a faculty-led lab. This approach allowed the students to adopt a researcher mindset beyond satisfying course requirements.

#### Domain 2: Personal Gains Related to Research

Students reported significant gains (mean of 4.38, all above 4.00) in this domain. The highest scoring items were *confidence in my ability to contribute to science* and *understanding what everyday research work is like, reflecting* the implementation discussed above. Significant gains in confidence, self-efficacy, independence, collaboration, and patience (specifically with pace of research) were reported congruent with results from the previous domain.^33^ The lowest scoring item (*discussing scientific concepts with others*) still is in the ‘good gain’ range. Scientific communication is one of the emphases of this CURE. Throughout the semester, students worked on several iterations of their final paper within the writing workshop (see Supporting Information - *Semester-long Writing Workshop: Improving Scientific Writing*). While this score reflects success, it also signals an opportunity for improvement, particularly in helping students become more confident in sharing concepts to a broader audience.

#### Domain 3: Academic Skills

An overall moderate gain (mean of 4.01) is reported in this domain, covering a broad range of research-associated skills. Of note in this domain are gains in *preparing a scientific poster* and *conducting database or Internet searches*. A university librarian helped with database searches and use of *SciFinder* as they plan for their research.^34^ The majority of the students have not presented in any conference prior (see also Figure 3 and corresponding discussion), so the required end-of-semester presentation is the first time they were involved in preparing a poster. This is part of the communication workshop, although reported gains in *making oral presentations* (3.50, the lowest in this domain) indicate an opportunity for improvement. Students presented their work in a symposium and to the instructors, as well as submitted an ACS-formatted manuscript, reflected in gains for *writing scientific reports or papers* (4.13). Good gains are reported for other skills with scores congruent with expectations for the course. Skills, such as using statistics or working with computers, were not part of the design of the current CURE and reflect limited opportunities rather than a lack of progress.

#### Domain 4: Changes in Attitudes and Behaviors

The final domain focuses on changes in student attitudes and behaviors as researchers showcase meaningful increases in self-efficacy for conducting research. Students reported feeling more engaged in real-world scientific work, thinking creatively about their projects, and feeling responsible for the outcomes of their research. The lowest score in this domain, and in the entire survey, is item 4.7, *Interact with Scientists from Outside Your School* (2.00). While interaction with the scientific community is indeed beneficial, this CURE did not have significant opportunities for this. The rest of the items have good gains (excluding item 4.7, the mean is 4.20). *Feeling responsible for the project* scored the highest in the domain (4.63).This again reflects and aligns with reported gains discussed in *Domain 1*. A comparison of the URSSA results reported here, with a caveat that our cohort is small (*n* = 8), to those by Weston (2012) shows that students in SynZyme reported higher mean gains in *Thinking and Working Like a Scientist* and *Personal Gains Related to Research* (Figure 1).^35^ This provides evidence of the efficacy of the implementation of the CURE, including its strengths and areas of improvement.

Overall, the URSSA results demonstrate that SynZyme CURE effectively supported the development of the students as researchers and *bona fide* members of the scientific community, with gains associated with confidence, capability, and independence.

### Insights from the Student Assessment of Their Learning Gains (SALG) Instrument

To support URSSA, which would not be enough to gauge the overall experience due to the small sample size, SALG, the master survey, was also used to understand student learning gains and motivations.

#### Quality of Research Experience

Quality of research experience depends on several factors including mentorship, collaboration, autonomy, and time commitment (Figure 2). Participants reported an average rating of 3.50 out of 4.00 (Likert scale, 1–4) for their overall experience. Students felt that they spent meaningful time doing research (3.63) and rated their *working relationship* and *amount of time spent with research mentors* good (3.50 and 3.00, respectively). This reflected the philosophy of providing the students freedom and an opportunity to take full ownership of their project. The mentors were hands-off and prioritized high-impact interactions (e.g., discussing viability of student ideas), rather than managing aspects of the project (e.g., suggesting conditions to try). Lab meetings also allowed the pairs to talk to each other and engage in collaborative discussions. This has enabled transfer of expertise (some students have prior experience with an instrument), learn from each other’s mistakes, and share ideas for aspects of the project that required optimization (e.g., assay conditions). Toward the end of the course, students were also able to compare results and make hypotheses for future designs, with some students performing substantial computational SAR and additional synthesis to test their hypotheses, in line with the iteration dimension of CUREs (see Supporting Information - *Example of Student Final Poster Presentations and Writing Workshop Module*). Students reported a lower score on getting advice about graduate school and career, as this was not necessarily an emphasis, and discussions were mostly student-initiated. This does point to an area of improvement, where gained skills can be linked to employability and articulating skills in preparation for the job market.^36^

**Figure 2:**
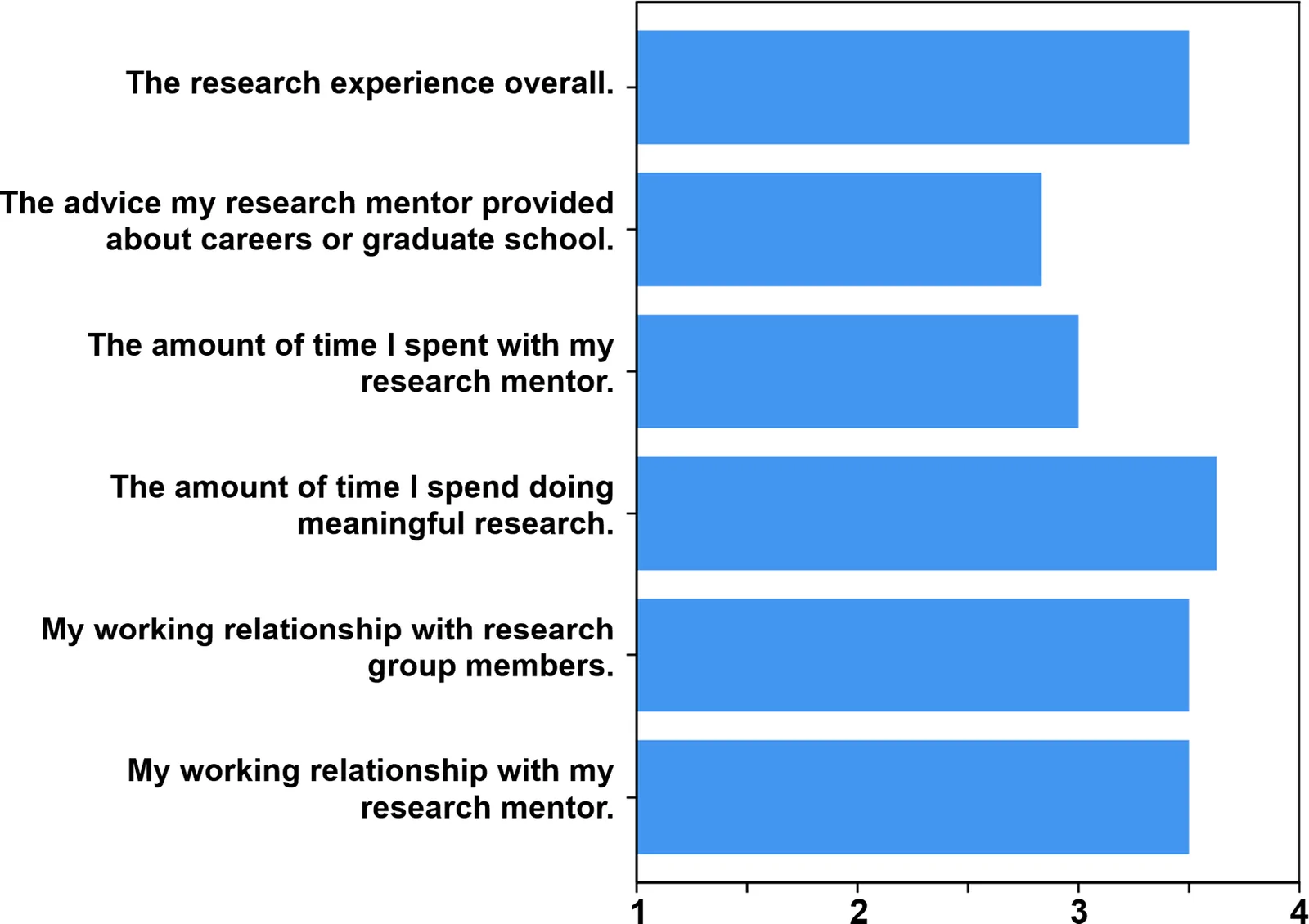
Evaluation of the quality of the CURE experience.

To better understand their experience, students were invited to provide comments, which highlighted gains (some covered above already) and the overall CURE structure. One student remarked, “*I really enjoyed this research project and the independence of it*.” Another elaborated on the structure of the course, stating, “*Overall, the research experience was extremely positive. We were given the opportunity to work independently, which encouraged us to take ownership of our tasks and manage our time effectively. This created a strong need for consistent communication between my team member and I, helping us stay aligned and ensuring smooth project progression. With greater responsibility placed on the us* [sic], *we became more aware of how our individual contributions affected the overall success of the project. Mentors/Professors provided helpful advice and guidance when needed, but maintained a largely hands-off approach that allowed us to troubleshoot, make decisions, and learn through direct experience. I really enjoyed it*.”

#### Scientific Communication and Research Milestones

Participants have varying levels of prior research experience (Figure 3). One of the aims of the CURE is to give students a chance to achieve some milestones including communicating their research. The course required students to prepare and orally present a scientific poster. This was presented in front of an audience of their peers, including other undergraduate students and graduate students, as well as faculty in a college-wide symposium. Most upper-division courses require student presentations, but most students, especially those not yet involved in faculty-led research, are unlikely to have presented in professional conferences. At the time of the survey, no students had authored or coauthored any manuscript nor had they won awards relating to their research. Outcomes expected from this CURE include scientific publications given the novelty of the molecules and protein targets. It is also important to note their intent on sustaining participation in scientific communication and professional development, with a few students indicating interest in presenting their research or pursuing publications.

**Figure 3:**
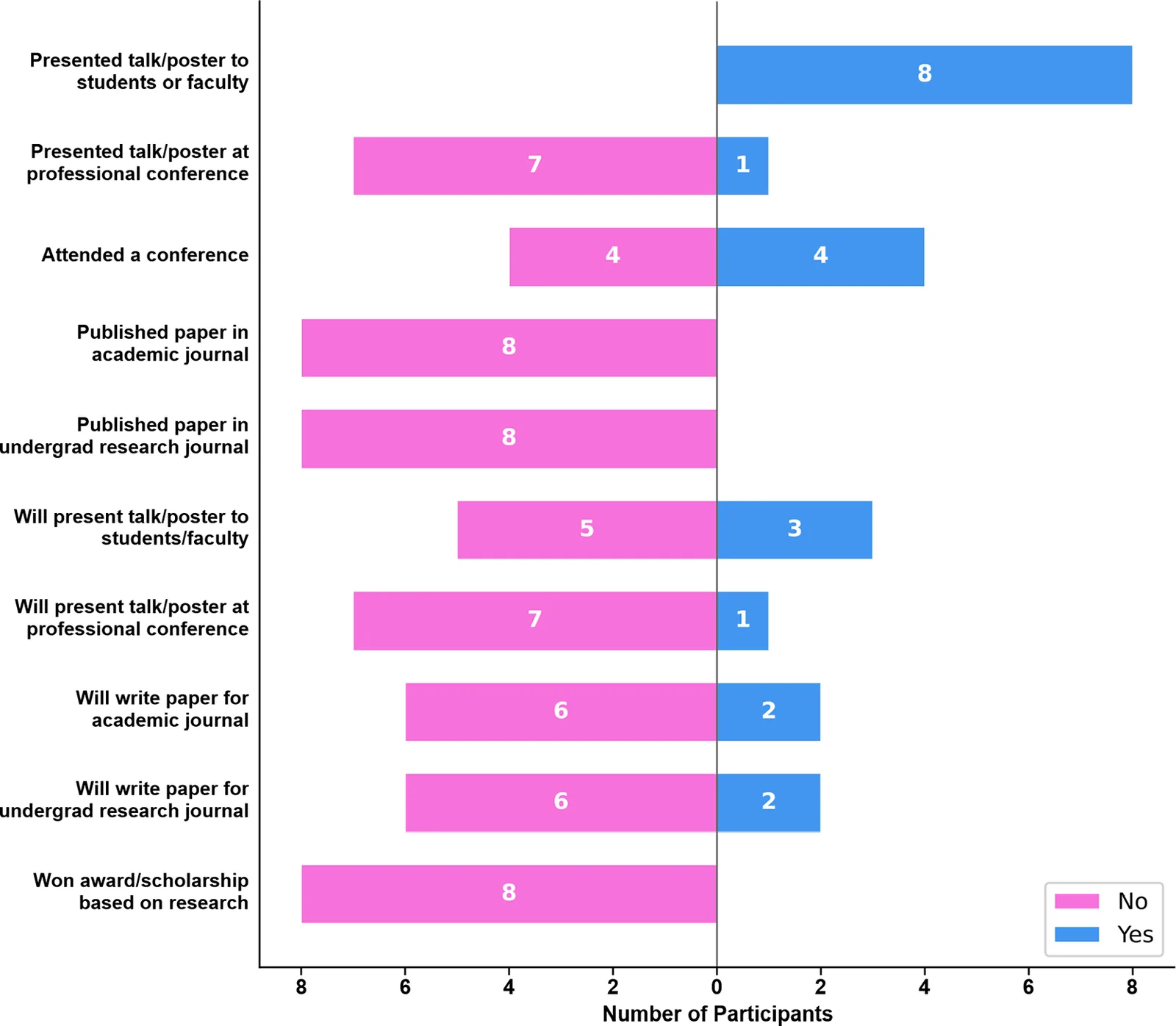
Student experience in scientific communication and prior research milestones.

#### Effects of Research Experience on Postbaccalaureate Plans

There is evidence that participation in CUREs correlated with continued and sustained interest in pursuing graduate education.^37,38^ We evaluated postbaccalaureate plans of students enrolled in the CURE, all of whom are chemistry/biochemistry juniors/seniors (Figure 1, Supporting Information Box 1). It is notable that 100% of the students indicated that they are more likely to *work in a science lab* and *enroll in a master’s program in science, mathematics, or engineering* after participating in the CURE compared to before. Pursuit of PhD programs is also rated highly (3.63).

To get a better picture of how their participation affected their plans and intentions after graduation, an open-ended question on their intended degree and intentions before and after participating in the CURE was asked (see Supporting Information Box 1). These responses support and clarify the results. There is evidence that the CURE contributed to their thinking about their career path, which appears to be adjacent to working in a research laboratory in some capacity (Figure 4). The responses also recapitulate gains discussed above, including independence and confidence in the lab. The responses are congruent with those observed in other CUREs.^12,37^

**Figure 4:**
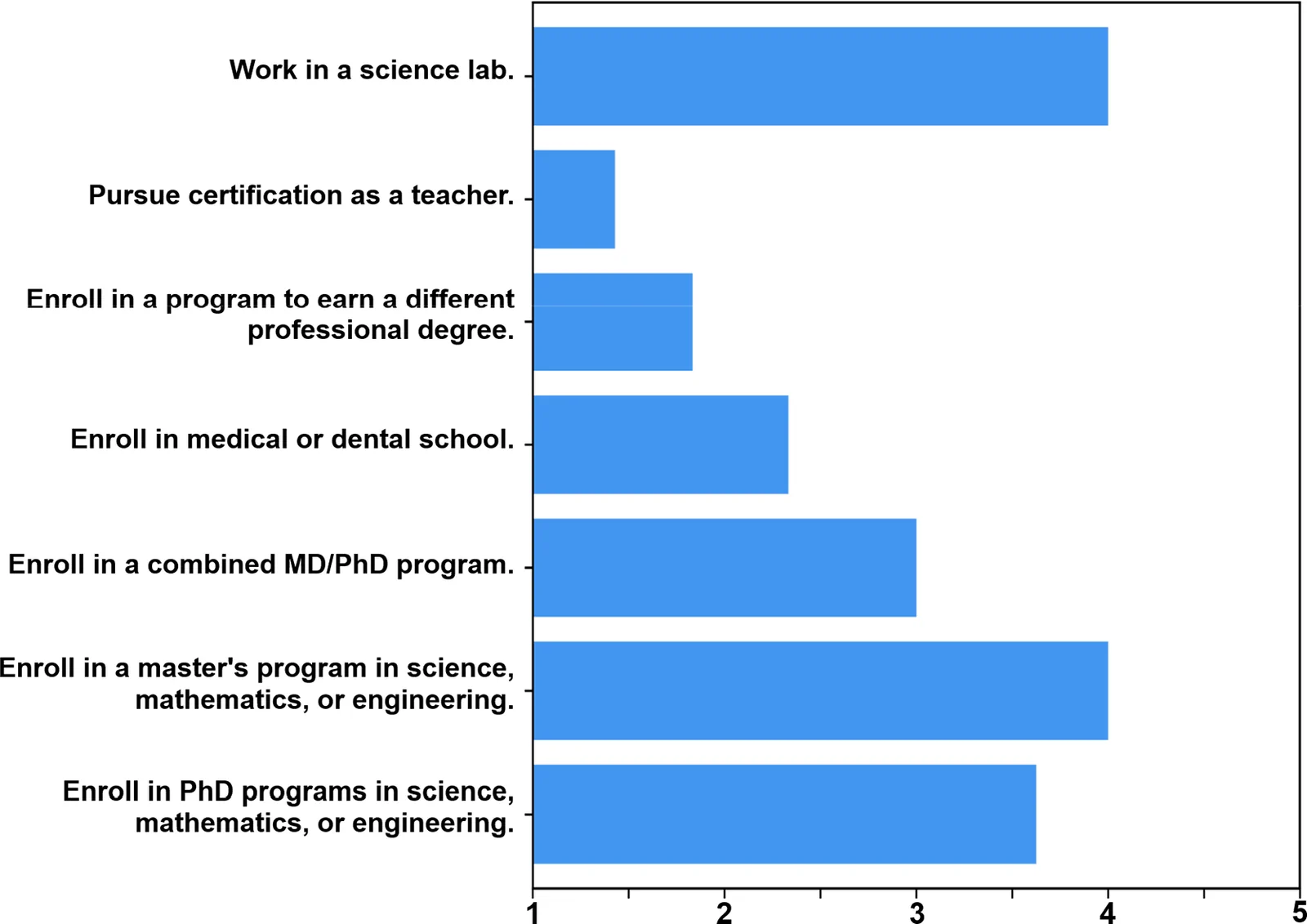
Effect of CURE experience on career prospecting.

Students self-reported gains in academic and career preparedness associated with the CURE (Figure 5). They report participation in research through SynZyme has either clarified or confirmed their field of interest. Furthermore, they reported gains in preparedness for advanced coursework, graduate school, and to a lesser extent, a job. These are congruent with their career plans and how the CURE has affected their goals and the way they think about their goals (Figures 4 and Supporting Information Box 1).

**Figure 5:**
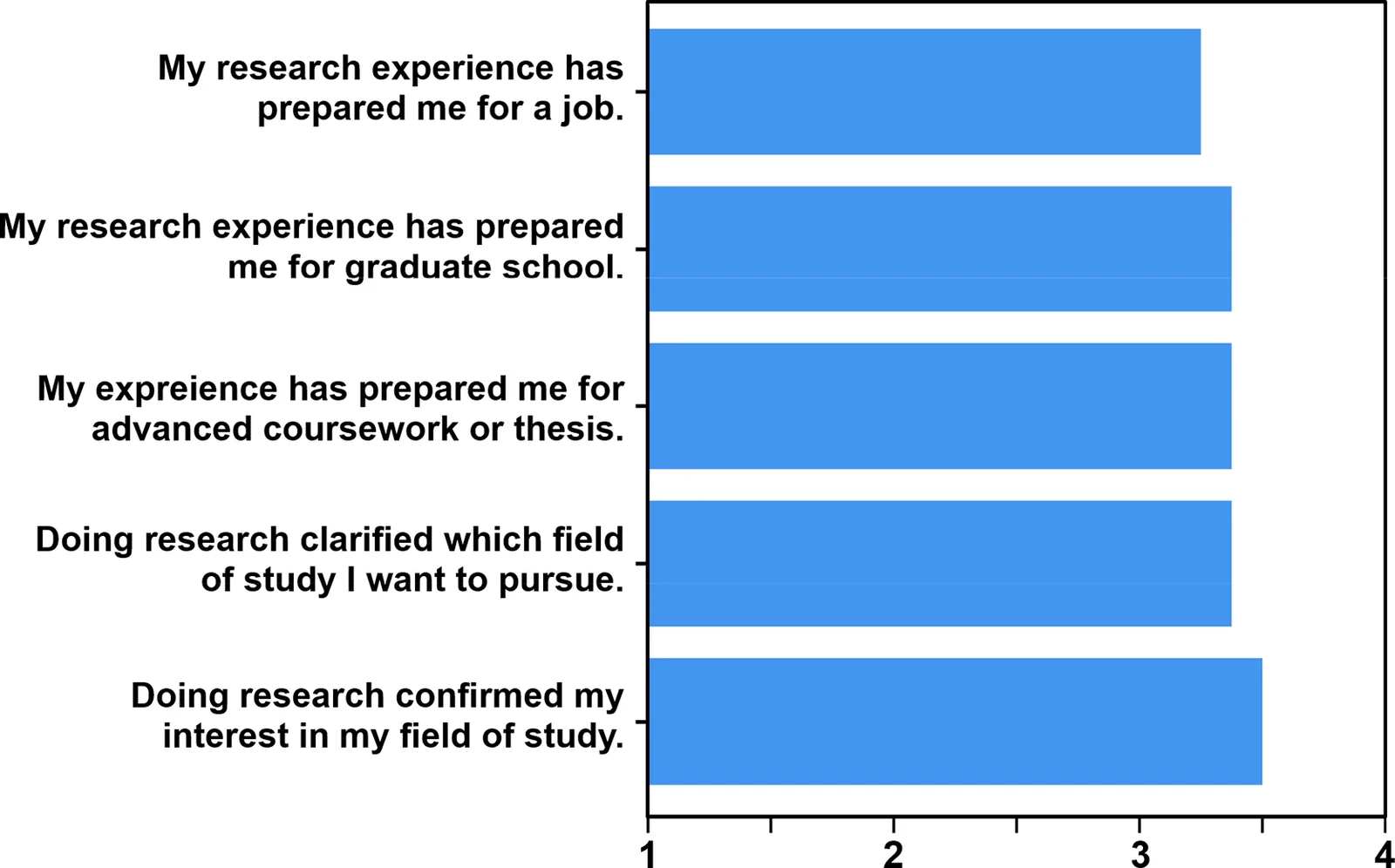
Effect of CURE experience on academic and career preparedness.

#### Student Satisfaction with the Program

Responses to a satisfaction survey were positive with a mean score of 3.74 out of 4.00 across all survey items (Figure 6). Support in terms of their partners, mentors, assisting graduate students, and availability of information was scored very highly. Clearly, these relationships, peer, near-peer, and faculty mentorships, are valuable components, simulating the dynamics of a research group. Accessibility and availability of laboratory equipment during the semester scored the lowest in this area (3.38). Infrastructural limitations, unfortunately, are a major obstacle even in faculty-led research. This underscores the importance of investment from the college or university level in instrumentation that can be used by undergraduate researchers.

**Figure 6:**
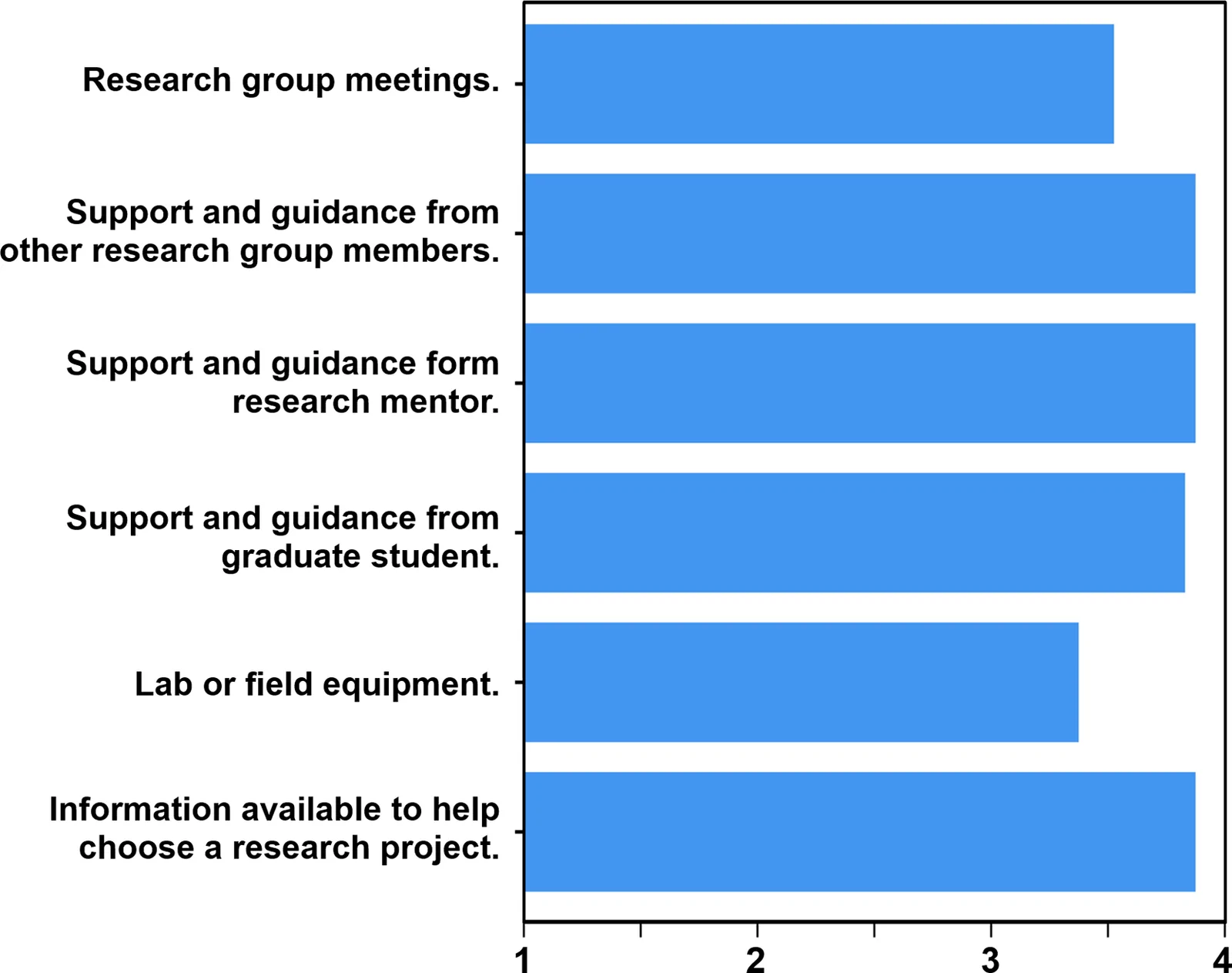
Student evaluation of their satisfaction with the CURE implementation.

#### Source of Information for Other Research Opportunities

CUREs lower the barrier to entry to research and enable faculty to engage more research students. We surveyed participants to understand how they learn about campus research opportunities (Supporting Information Figure 1). Students most commonly reported learning about them through classes rather than through academic advisors or institutional announcements. This confirms that CUREs can become a bridge for students to begin engaging in long-term, faculty-led research. In the case of this CURE, however, this is not likely, given that the majority of the students are already seniors.

#### Motivation of Students for Pursuing Research Opportunities

The underlying motivation of students for participating in research can dictate the type of experience that they have. A varying level of motivation was evident in the participants. Highly motivated students pursued additional questions, supplementing their training and increasing the impact of their outcomes. We surveyed our students to understand their motivations (Figure 7). A majority of the students indicated that they were interested in pursuing research to explore their interests, clarify the field they want to study, and engage an intellectual talent and not necessarily working with a particular faculty. Students indicate that they pursue research experiences to enhance their résumés, but only 50% do so to secure good recommendation letters. These responses suggest that these students view research as an opportunity to challenge and learn more about themselves as scientists. This reflects that intrinsic interest in doing science/research and improving as a scientist is a primary factor in their decision-making.

**Figure 7:**
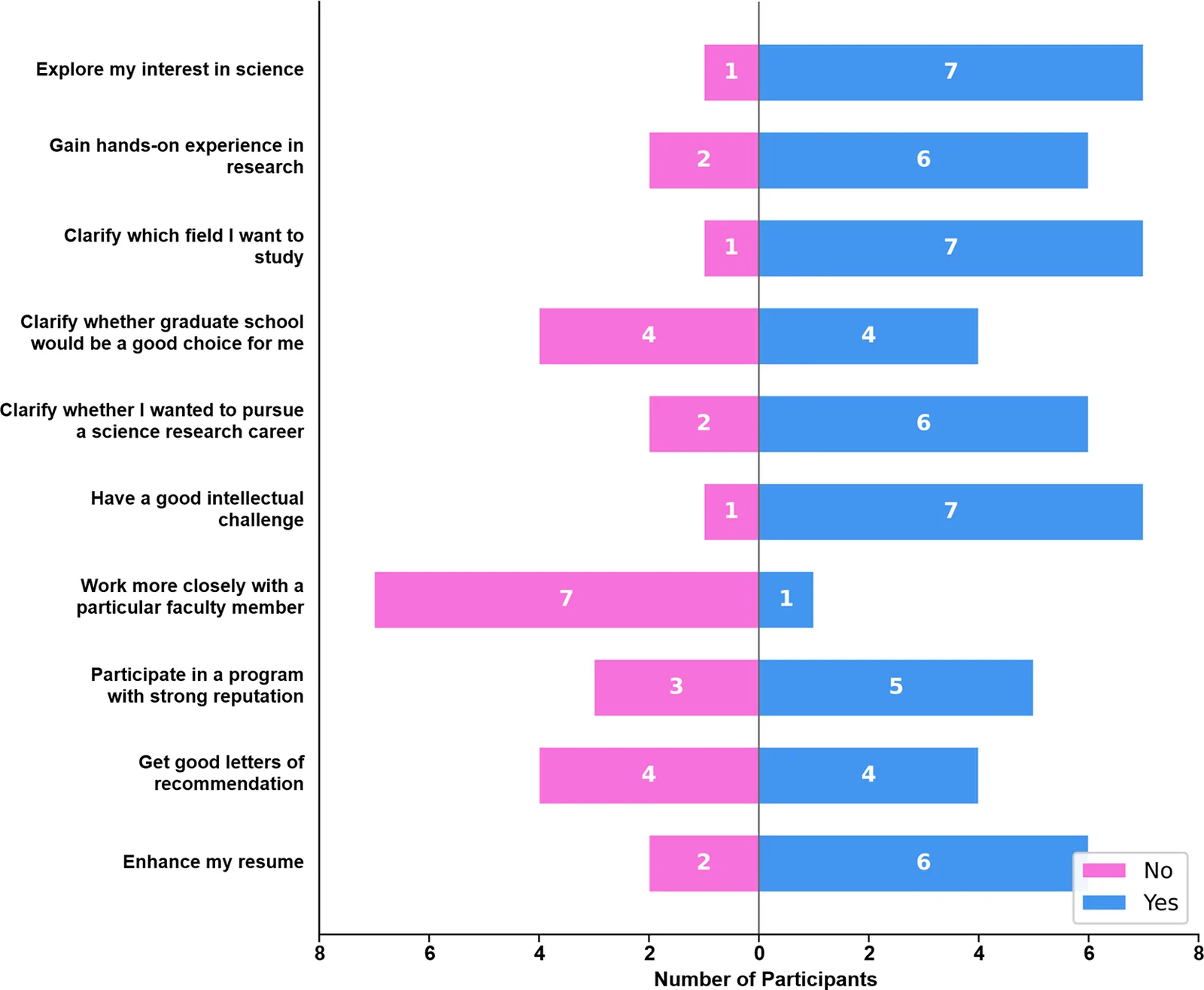
Different motivations of students for participating in research.

#### Student Commitment to Research

Some students reported engagement in faculty-led research before the CURE. A few are involved in long-term research, while some participated in summer research. This indicates, for this cohort, an inherent interest in research. Students devoted varying amounts of time to conducting their CURE projects. This ranged from a few hours a week, during scheduled time, to more intensive commitments, including working extra hours for troubleshooting or repeating experiments and pursuing additional goals. Some students committed 15+ hours in some weeks to learn, run, and analyze simulations, to synthesize and characterize additional compounds, or to perform a three-step (including tag cleavage and size exclusion) rather than a one-step purification. This reflects differing immersion levels and heterogeneity in the student motivation and interest. They also reported regular interaction with the mentors (see also Figure 6), engaging in weekly discussions outside scheduled hours (see Supporting Information - Table 2).

#### Additional Student Feedback on the CURE and Reflections on UG Research

Students were given a chance to provide additional details on their experience focusing on their thoughts to improve the CURE (Supporting Information Box 2). Feedback largely concerned access to instrumentation (see also Figure 6). While unrelated to the CURE, it is worth noting that availability of paid positions also contributes to lowering the barrier-to-entry to longer-term research. CUREs provide students an opportunity to participate in authentic research within the curriculum, whereas other opportunities may be associated with opportunity cost. Through end-of-semester student rating of instruction, students were asked to identify components that contributed the most to their learning. One student reported that “*the CURE worked well with their schedule*,” while another indicated they “*benefited from the hands-on experience and independence the course offered*.” Overall, feedback reflects the success of this CURE.

### Positive Outcomes from the Course

SynZyme was a response to declining enrollments in our upper-division courses, necessitating cross-listing of instrumental and bioinstrumental analysis. It was implemented to provide an interdisciplinary experience that bridged unique needs within these courses and enhanced student engagement in an upper-division course.

Five triazoles were synthesized, yielding five publication-quality structures. Participants purified and optimized assays of the target PTPs, providing a foundation for future structural studies and chemical screening. Initial inhibition assays of the triazoles indicate that two are potential hits on one of the PTPs, leading to two full-fledged projects beyond the CURE. Several triazole designs were also identified as two groups pursued extensive computational structure–activity relationship studies. Genuine engagement and collaboration are evident from the student responses. Overall, SynZyme demonstrated the viability of an interdisciplinary experience that bridges organic chemistry and biochemistry. As an upper-division course, this allows the students to draw from prior curricular experiences while engaging in research that has broad relevance and tangible real-life impact.

Participants had extensive scientific communication experience, including a final course presentation and an ACS-formatted manuscript. SynZyme culminated in a college symposium where all groups presented a poster to an audience of 60+ undergraduate, graduate students, and faculty. Participants who remained enrolled after the semester also chose to present at an additional university-wide conference.

Participants recognized other intangible skills and traits that improved during the course of the intensive project, including time management, ownership of their project, and autonomy. They developed a better appreciation of the concepts discussed in the lecture, which was aligned with the CURE through real-world applications of instrumentation. As of this writing, two of the students have transitioned into the MS program, one joined the BS-to-MS track, and two have graduated. Five remain involved in faculty-led research.

### Limitations and Future Directions

SynZyme was implemented at an R2/PUI and delivered by two instructors from separate campuses. The course is offered on one campus, but necessary expertise and equipment required coordination between two campuses. The outcomes were strongly positive and demonstrate the efficacy of integrating organic chemistry and biochemistry within an instrumental analysis framework. Logistical constraints emerged during the pilot. Coordinating a research-intensive course across two campuses introduced challenges related to travel, scheduling, and the distribution of instructional responsibilities. Time limitations within a single semester also restricted the depth of experimental work, particularly for students who wished to pursue extended synthesis, purification, or enzymatic assays. The nature of this project, as a drug discovery program, lends to a considerable opportunity for iteration by improving inhibitor response. Time constraints in a one-semester course, however, preclude this, but as demonstrated by some of the groups, computational SAR is doable and is equally enriching. Students frequently requested additional access to laboratory space and instrumentation. The sample size (*n* = 8) limits generalizability of the findings, and compelled us to utilize the SALG master survey along with URSSA.

Future iterations may benefit from strategically dividing instrument access among groups such as by implementing a staggered scheduling strategy. Incorporation of higher-throughput alternatives such as using batch purification instead of an FPLC or colorimetric assays using portable visible-light spectrophotometers also helps in addressing instrumentation constraints. As the class is embedded within an instrumental analysis course, practical’s on the FPLC will need to be included in the schedule. This gives the students an opportunity to compare both approaches. Confidence in presentation skills can be improved by incorporating a midterm oral presentation to the class. Additionally, inviting external speakers as guest lecturers during the term will give the students an opportunity to interact with the broader scientific community.

Scaling the course for larger class sizes will require restructuring. In addition to alternative methodologies and planned schedules, support from teaching assistants and optimized protocols will help a larger class. The instructional model also shows promise for integration into other upper-division courses, such as advanced organic chemistry or advanced biochemistry, and may be effective when implemented as a two-semester sequence, bridging the final course in an organic series and the first of a biochemistry series. Introducing docking earlier in the semester, particularly in the design stage, could enhance the experience.

Finally, the nature of the project makes it amenable for expansion, whether by improvement of previous inhibitor hits or investigation of protein tyrosine phosphatases from other bacteria. This approach can also be adopted to other therapeutic areas such as antifungals and anticancers and incorporate evaluation of inhibitor selectivity, especially given conservation among PTPs. Supported by training in various chemical and biochemical instrumentation, the CURE also equips chemistry and biochemistry majors with skills necessary for solving problems of the 21st century. Student motivation remained exceptionally high throughout the semester, with many expressing interest in continuing projects beyond the course.

## Conclusions

SynZyme demonstrated the successful engagement of undergraduate students in authentic scientific discovery. Participants synthesized and characterized 1,2,3-triazole-based compounds and identified inhibitors of bacterial phosphatases for antimicrobial development. Participants will be coauthors in at least two manuscripts. We also anticipate two new research projects beyond the CUREs. Data from the URSSA survey report gains in thinking and working like a scientist and personal gains related to research, comparable with published benchmarks. These are supported by student responses, highlighting autonomy, ownership, responsibility, and intrinsic scientific curiosity as drivers of engagement. There is an indication of success for the implementation of this CURE, including strong self-reported gains, tangible research outcomes, and even sustained research engagement. Students recommended increased access to instrumentation and additional time to complete the experimental work. Overall, the student responses suggest that the CURE has achieved the primary goals of increasing student engagement and combining synthetic chemistry and enzyme biochemistry (SynZyme) supported by an overarching antimicrobial discovery theme. The model should be replicable, expandable, and suitable for use within a chemistry, biochemistry, or biology curriculum.

## Supplementary Material





## Notes

Course Syllabus, Course Schedule, Recruitment Email, Informed Consent, Laboratory and Instrumentation Details, Student Demographics, Survey/Instrument used (PDF, DOCX)
