# Supplementary material for: Development of Interdisciplinary SynZyme CURE: A Bridge between Organic Synthesis, Protein Biochemistry, and Drug Discovery
Source: J Chem Educ. 2026 Aug 20;103(9):4967–77. doi: 10.1021/acs.jchemed.6c00350 (PMC13564351; doi:10.1021/acs.jchemed.6c00350)
Supplement: Supplementary file 1 [file ed6c00350_si_001.pdf]

Supporting Information

Development of Interdisciplinary SynZyme CURE: A bridge between organic synthesis, protein biochemistry, and drug discovery

Scott Westervelt,[1] Grace M. Bennett,[2] Mark V. C. dela Cerna[2]*, Shainaz Landge[1]*

[1]Department of Biochemistry,Chemistry, and Physics, Georgia Southern University, 521 College of Education Drive, Statesboro, Georgia, 30458, USA

[2]Department of Biochemistry,Chemistry, and Physics, Georgia Southern University, 11935 Abercorn Street, Savannah, Georgia, 31419, USA

*Corresponding authors: mdelacerna@georgiasouthern.edu and slandge@georgiasouthern.edu

Table of Contents:

Syllabus ……………………………………………………………………………..…… 3

Schedule ……………………………………………………………………………..…… 9

Recruitment Email ……………………………………………………………...……..……11

Informed Consent……………..………………………………………………………………12

Details on Practical's and Instrumentation……………………………………...….……14

Sample Course Materials (Lab Safety and Lab notebook)…………….……….….…18

Semester-long Writing Workshop: Improving Scientific Writing…………….…..……19

Student Final Poster Presentation Examples ……………………………….…..……20-21

Table 1: Student Demographics……………..……………………………………...……22

Figure 1: Research Opportunities on Campus…………………...…………...……22

Table 2: Student Commitment to Research ……..………………………………...……23

Student Assessment of their Learning Gains (SALG; Master Instrument)
Undergraduate Research Student Self-Assessment (URSSA) item 1-4……………24

Student Assessment of their Learning Gains (Adapted) - remaining items…...……25

Box 1: Responses to Open-Ended Questions ……..……………….……………........30

Box 2: Additional Student Feedback ……….……..………………….……………........31

Fall 2025 Course Syllabus

Bioinstrumental Chemistry SynZyme CURE 2025

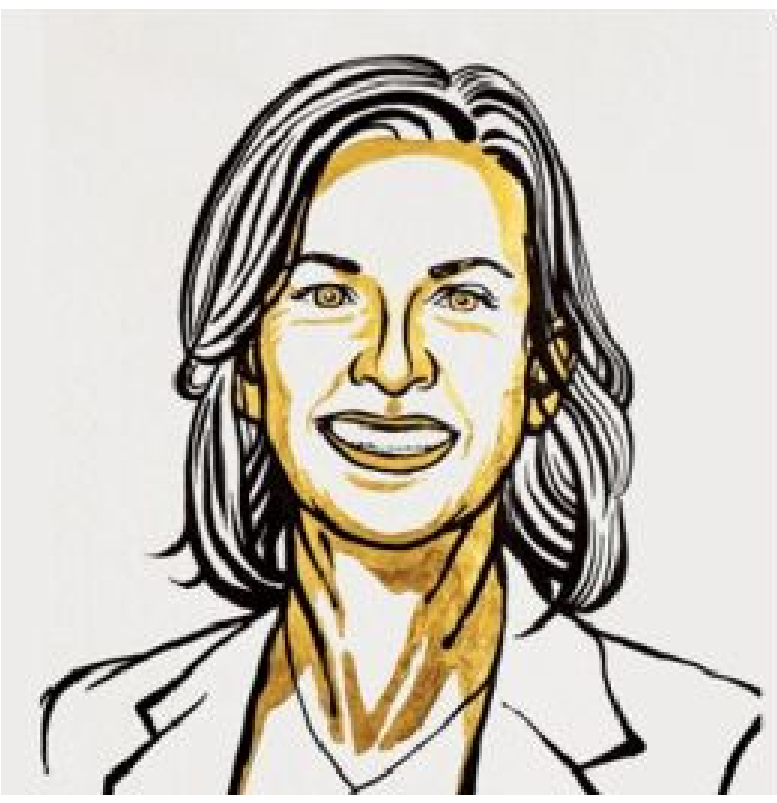

"All scientists, regardless of discipline, need to be prepared to confront the broadest consequences of our work—but we need to communicate its more detailed aspects as well."

– Jennifer Doudna
2020 Nobel Prize in Chemistry
"for the development of a method for genome editing"

COURSE INFORMATION
Prerequisite: CHEM2100: Analytical Chemistry
Cross-listed as CHEM3100 Instrumental Analysis
Time: 12:30 – 3:20 pm (M); 12:30-1:45 pm (T/TR)
Science Center 2016

INSTRUCTOR INFORMATION

Dr. Mark Vincent C. dela Cerna (he/him)
mdelacerna@georgiasouthern.edu
Office: 912-344-2507
Office: Science Center 2012 (Armstrong)
Student Hours: By Appointment, Monday 11:00 am – 12:30 pm

Dr. Shainaz Landge (she/her)
slandge@georgiasouthern.edu
Office: 912-478-1883
Office: Nursing/Chemistry 3213 (Statesboro)
Student Hours: By Appointment, Tuesday/Thursday 11:00 am – 12:00 pm

COURSE DESCRIPTION
This course will introduce theory and practice of instrumentation in chemical and biochemical sciences. Students will learn about the theory and practice of key instrumentation in lectures and directly apply them in semester-long research that combines organic synthesis, protein biochemistry, and drug discovery. The course will cover best practices on sample preparation, use of instrumentation, data analysis, and troubleshooting combined with the necessary theory.

OVERVIEW OF COURSE METHODOLOGY

The objective of this course is to enable students to gain mastery of instruments used in chemical and biochemical analysis, with an emphasis on small molecules and proteins, and their interactions. The course is supported by a semester-long, research-based laboratory that tackles real-world problems involving organic synthesis, drug screens, and protein-targeted drug discovery. As such, there is emphasis on best practices, experimental design, hands-on use of instrumentation, data analysis, and troubleshooting. Whenever possible, classes will involve demonstrations and hands-on learning. Lectures will largely consist of independent study, guided discussions, and interactive elements such as recitations, demonstrations, and journal clubs. The course will offer a survey of modern instrumentation actively used in scientific literature, focusing on those available in the Savannah Campus of Georgia Southern. The journal club discussions will focus on the application of the techniques, expected results, limitations and strengths of the methods used, and potential alternative instrumentation. There will be a substantial laboratory component which will include a semester-long research-type project with a few training-focused sessions. Laboratory sessions will provide the students the opportunity to gain expertise in operating various instrumentation available on campus.

COURSE LEARNING GOALS

After taking this course, students will be able to:

1. identify and describe a variety of analytical instruments, including their uses, capabilities, and limitations.
2. describe the fundamental principles of spectroscopy and chromatography.
3. evaluate different instrumental methods based on their suitability for certain applications and relative performance.
4. analyze and present data obtained using various analytical instruments and use the information to address specific questions.
5. read and understand scientific papers and relate the information to their peers and a broader audience.
6. demonstrate the ability to productively participate in group situations and engage in effective teamwork and collaboration.

REQUIRED TEXT

Students **are not required** to purchase any text. Any required reading materials will be provided in class. However, students may use Harris' Quantitative Chemical Analysis and Lottspeich's Bioanalytics as references. Both are available in the Lane Library. While purchase of text is not required, students are required to come to class prepared. Students are expected to have read *any* reading provided ahead of time, including book chapters, tutorials, and primary literature. All required reading materials will be provided at least two days ahead of the lecture in which they will be covered. Students should expect that the contents of these reading materials are going to be part of any assessment (quizzes, exams) whether or not they are covered in the lecture.

TECHNOLOGY POLICY
By default, students are not allowed to use any electronic devices during any of the lectures. Students are, however, expected to bring a calculator to every class and laboratory session. Students are allowed to use any piece of technology during the laboratory sessions as long as they are not distracted from their work. Students are encouraged to use Excel or python (or any tool) to visualize and analyze their data collected from the laboratory exercises and for their projects.

GRADING
Letter grades are assigned to final grades as follows:

**A**	90.0% or higher
**B**	80.0 to 89.9%
**C**	70.0 to 79.9%
**D**	60.0 to 69.9%
**F**	lower than 60.0%

Non-graduating students with a grade of 90.0% prior to the final are exempt from the final exam and will receive an A for the class. These "pre-final" grades exclude any extra credit, if any are provided throughout the semester.

Note: If either the laboratory or lecture grade, individually, is less than 60%, you will get an automatic F in the course. That is, you need to pass both sections to pass the course.

'LIFE HAPPENS' POLICY
Attendance and late submission policies will be strictly enforced. However, we recognize that *life* happens. If at any point, an event truly disrupts your progress in this course, let me know right away. Certain issues can compound over the course of the semester and by keeping me in the loop, we will be able to make accommodations for you that will, hopefully, provide you with the *opportunity to still succeed* in the course. These policies are in place to incentivize students to be responsible for their work and reward those who work hard. Unique circumstances, however, should not rob any student of a fair chance not only to attain a fair grade, one that is not deflated by their circumstances, but also to truly learn and get the most out of the class. You are a human first and a student second. That said, some requirements (see ***Course Requirements***) cannot be made up and any missed work will result in a zero in that activity.

COURSE REQUIREMENTS
The final grade will be calculated based on scores for each of the following components.

Quizzes (15%) - Quizzes will be given in the first 5-10 minutes of *every* class. Quizzes will not be made up. They will cover topics already discussed and assigned reading.

Exams (30%) - There will be a total of two major exams throughout the semester covering theory and practice. Exams will cover whatever is discussed in the lecture, covered in the lab, and assigned as reading assignments. These exams are scheduled ahead of time. If you have any conflict, let me know during the first week. All exams are comprehensive.

Comprehensive Final Exam (15%) - The comprehensive final exam will be a multiple choice exam scheduled by the University. Any conflict must be dealt with immediately.

Laboratory Performance (40%) - This component includes any activity that is done outside the lecture, including the semester-long research project and all outputs in the lab, broken down into the following:

- **100 points** - Research paper (Group)
- **100 points** - Writing Workshop Activities (Individual)
- **100 points** - Final presentation (Group)
- **50 points** - Laboratory notebook (Individual)
- **100 points** - Research-based homework assignments (Individual)
- **50 points** - Laboratory behavior and attendance (Individual/Group/Class)

ATTENDANCE AND POLICY ON MISSED/LATE WORK/SUBMISSIONS

<u>Students are expected to attend all sessions</u>. Late work will still be accepted but the grade will be capped at 15% less than the class average. No more work will be accepted during the last week of class (unless it is the original deadline). If a student misses any work, other than quizzes (no make up), they can discuss the case with the instructor. Possible outcomes will depend on the case and may include dropping the activity, getting a zero, or a make-up activity (similar, not the same). It is the student's responsibility to initiate any such conservation. By default, if work is not submitted, it will receive a zero.

PLAGIARISM AND CHEATING <u>will not be tolerated in this class</u>. This includes unethical use of any AI-based tool. In addition to a report to the appropriate deans and student court, any offense will result in an automatic F in the course.

TEACHING PHILOSOPHY

One of the ways that will contribute to your success in this course is ensuring that you are communicating to us and your classmates. We approach every class that we teach thinking that *<u>all of you want to succeed,</u>* and we want that for you as well. In fact, we want you to succeed not only in this course, but in your other courses and activities as well. Note, however, that success does not necessarily mean high grades – so our goal is not to help you get high grades but to help you learn the most from the class. High grades are usually a by-product of the learning process. In courses we teach, you can succeed by engaging, participating in discussions, and asking questions. We will always appreciate you being proactive in your learning. If not during class, ask these questions during student hours! These hours are reserved for you so take advantage.

Overall, at the core of my teaching philosophy is the idea that we are collaborators – we are working towards your success in understanding the concepts we will cover in this

class together. *We are as invested in your learning as you are – and the more you invest in it, the more we will too.*

We consider this classroom to be a place where you will be treated with respect and where you will treat others with respect. Individuals of all ages, backgrounds, beliefs, ethnicities, genders, gender identities, gender expressions, national origins, religious affiliations, sexual orientations, abilities, military status, and other visible and non-visible differences, are all welcome in this class. All members of this class are expected to contribute to a respectful, welcoming, and inclusive environment for every other member of the class. Multicultural and intercultural awareness and competencies are key leadership skills, which will serve you in this classroom, educational environment, and in life. If you feel we are not providing an inclusive environment in the classroom setting, please let us know what we can do for you. Disrespect of any form will not be tolerated in the class.

COMMUNICATIONS POLICY

We encourage active communications all throughout the semester – as said in the teaching philosophy, this is a collaboration. This includes asking questions in or before/after class, dropping by the office during student hours, or making an appointment for individual or group consultations. Outside student hours, email is the preferred mode of communication. Students should use the following subject line so we can respond in a timely manner: **BIOINSTRUMENTAL: <subject>**. For example, **BIOINSTRUMENTAL: question about mass spectrometry**. If you feel it is urgent, put **URGENT** in the subject line.

UNIVERSITY ATTENDANCE POLICY (FIRST DAY)

University policy requires all students to attend the first meeting of all classes for which they are registered. Faculty must verify first day attendance, and any student who does not attend the first day of classes will be dropped from that class.

A NOTE ON ILLNESSES

Georgia Southern University wants you to take appropriate precautions for your health as well as the well-being of your classmates. If you become ill during the term, please contact me immediately. We will work through what you will need to do, to either continue working in class or make up work that might have been missed during your absence. If you have an illness that would result in an extended absence, you will need to contact the Dean of Students office. In the event of serious illness, injury, or extenuating circumstances, the DOS office will notify professors at your request. For more information about the DOS, follow this link:
https://students.georgiasouthern.edu/dean/.

CODE OF STUDENT CONDUCT

Students are responsible for knowing and following all policies laid out in the current Georgia Southern University Code. All graded assignments such quizzes, exams, and homework are to be the student's own work. Violations of the university Code of Student

Conduct including, but not limited to, cheating and plagiarism, are taken very seriously. Any violation of this policy will become part of the student's permanent educational record. The student may review the Code of Student Conduct via the link below : https://students.georgiasouthern.edu/conduct/student-code-of-conduct/

SEXUAL MISCONDUCT AND EQUAL OPPORTUNITY

Georgia Southern University is dedicated to providing a safe and equitable learning environment for all students. Discrimination, sexual assault, and harassment are not tolerated by the university. Students are encouraged to review the policy found at https://president.georgiasouthern.edu/eeo-titleix/ and may report any incidents using the form on that website or sending an email to eeo-titleix@georgiasouthern.edu.

ACCOMMODATIONS

Georgia Southern University is committed to providing reasonable accommodations to students with documented disabilities as required under federal law. This includes ADD or ADHD, autism spectrum disorder, brain injury, chronic medical conditions, communication disorders, hearing loss, learning disabilities, mobility impairment, psychological disorders, visual impairment or temporary injuries. The purpose of disability accommodation is to provide equal access to the academic material and equal access to demonstrate mastery of the material. If students need accommodations, they must contact the Student Accessibility Resource Center (SARC) who will provide them with an accommodation letter detailing approved accommodations. Disability accommodations work best starting at the beginning of the semester but can be approved and started at any point in the semester. Accommodations start at the time the accommodation letter is presented to faculty within reasonable timelines; accommodations are not given retroactively. SARC for the Savannah campus is located on the second floor of Memorial College Center and the phone number is (912) 344-2572.

CAMPUS CARRY

Georgia House Bill 280, commonly referred to as the "campus carry" legislation, took effect as of July 1, 2017. For more information on this new law (which amends O.C.G.A. § 16-11-127.1) and how it will be implemented on University System of Georgia campuses, please read http://www.usg.edu/hb280.

Tentative Schedule of Activities
SynZyme CURE 2025

Week	Focus	Instructor Notes
1	SynZyme Introduction and Orientation	Students to be introduced to the concept of a CURE and the broad goals of the SynZyme project.
2	Synthetic Chemistry Overview and Laboratory Preparation	Overview of the synthetic methodology to be introduced and students will choose their molecules. Assigned laboratory spaces to be organized and all reagents (for synthesis, media, and buffers) prepared.
3	Synthesis	Students will begin their synthesis and may proceed to characterization, including melting point and TLC.
4	Synthesis / Clean-Up	This week will serve as a buffer week for additional time to re-synthesize or perform clean-ups as necessary, some students can proceed to other characterizations.
5	NMR Spectroscopy (Statesboro)	Dedicate a week for NMR to use a higher field NMR available in the Statesboro campus. All NMR spectra were collected where applicable: proton, carbon, fluorine, phosphorus. All students should have compounds at this point and can collect all other required data.
6-7	Characterization and Protein Expression	Other characterizations including mass spectrometry, fluorescence, and UV-Vis spectroscopy will be done here. Students will also work with a research lab on protein expression. Crystallization should be set up at this point, if not yet done in prior weeks. (Note: expression not necessarily part of student responsibility as most have done this in BCHEM5201)
8-9	Protein Purification	Focus on FPLC and protein quantification and evaluation by SDS-PAGE. Those who need to re-express

		may also use this week. Flexibility critical on timing. Students with enough protein may do assays.
9-11	Functional Characterization and Assay Optimization	Week focuses on enzyme activity characterization. Students may use UV-Vis spectrophotometer or plate reader, depending on availability. All students must use the plate reader for final assay.
11-12	Inhibition Assays	Need flexibility due to limited instrumentation. Expectation is for students to have done at least one trial at the end of this period.
13	Binding Characterization	Focus on ITC and/or send samples out for protein NMR.<br><br>*Post-semester note: This week was instead used as a catch-up week as some students still needed some characterization and inhibition assays done. Other groups also chose to pursue additional experiments, including additional molecules (attempted) or computational work.*
14	SynZyme Wrap-Up	Debrief the students, and ensure everyone has all the data they need to proceed with their write-ups, presentations, and posters. This is also the final week that students can work in the lab for last-minute work and to clean up the spaces to wrap-up the semester.
15	Poster Presentation	Students present their work in symposium in collaboration with the Vertically Integrated Projects Program at Georgia Southern and First-Year Experience (FYE) Researchers.

Recruitment Email

Dear Students,

I am working on a Chemical Education research project to better understand students' experiences in this Course based Undergraduate Research Experience SynZyme BioInstrumental course and how it impacts on students' perceptions and research gains and its impact on student success.

We plan to use the information from this research to develop better skills, education strategies, and techniques to enhance how students can benefit in future CURE courses. I would kindly ask you to participate in an online survey for assessment.

The confidential survey will focus on your learning experiences in the CURE course. The survey will be open on the last week of course work (December). I will not have access to your individual responses until the end of the semester.

In any presentation of the results, we will not include any student name or any identifying details. If you provide answers, please do not include any names or details that can identify you, or your friends, relatives, roommates, or the individuals you are describing.

You must be 18 years of age or older and a current undergraduate student at Georgia Southern University to participate in this study.

If you are under 18 years of age and a high school student dually enrolled at Georgia Southern, please indicate so in the informed consent but do not complete the survey.

If you're interested in participating in this research, please click on this link. The hyperlink (will be inserted) will take you to an informed consent form, which will provide additional details about the study followed by survey.

Thank you so much!

Sincerely,

Dr. Shainaz Landge and the SynZyme CURE team

Appendix A

COLLEGE OF SCIENCE AND MATHEMATICS

DEPARTMENT OF CHEMISTRY AND BIOCHEMISTRY

Informed Consent
for
Synzyme CURE

My name is Dr. Shainaz Landge, an associate professor of chemistry here at Georgia Southern University.

Purpose of the research:
You are being asked to take part in a research study of how the Course Based Undergraduate Research Experience (CURE) involving synthesis and enzyme inhibition activities enhanced your student performance and attitudes.

The purpose of this research is to determine the impact of the SynZyme Course-Based Undergraduate Research Experience (CURE) on students' perceptions and research gains and its impact on student success.

What we ask of you:
If you agree to be in this study, your course grades and survey responses will become part of a large dataset which we will use to conduct our analysis; once you have completed the course, your data and participation will be completely anonymous. The majority of this study requires no time commitment from you other than that associated with normal course attendance and course lab activities.

Risks and benefits:
The risks of the study are minimal, and no more than you would encounter in your day-to-day life. We do not anticipate that you will benefit directly from participating in this experiment, but we hope to use the data acquired to benefit future STEM students at Georgia Southern University and beyond. If you are younger than 18, you will be excluded from the study

Incentive or compensation:
There is no incentive for participating in this study, and you will not be adversely affected in any way if you choose not to participate. Your participation in this study is completely voluntary, and you have the right to elect to discontinue participation in this study even after you have consented without penalty.

Confidentiality:
Your identity will be kept confidential to the extent provided by law, and your data will be anonymized before data analysis begins. Your non-anonymized data as it relates to the study will be seen by your mentors. When the study is complete, all non-anonymized data will be destroyed. Your name will never be used in any correspondence, report, or publication. This consent form along with your anonymized data will be kept for three years beyond the end of the study and then destroyed.

Whom to contact if you have questions about the study:
Dr. Shainaz Landge (912-478-1883 or slandge@georgiasouthern.edu).

For questions concerning your rights as a research participant, contact Georgia Southern University Office of Research Services and Sponsored Programs at 912-478-5465.

Statement of Consent: I have read the above information, and have received answers to any questions I asked. I consent to take part in this study.

Your Signature ___________________________________ Date ________________________

Your Name (printed) ____

Signature of person obtaining consent ______________________________ Date ___________________

Printed name of person obtaining consent ______________________________ Date _______________

You will be given a copy of this consent form to keep for your records. This project has been reviewed and approved by the GSU Institutional Review Board under tracking number #####.

Details on Practical's and Instrumentation

General Procedure for the Synthesis of Triazoles:[1]
In a 10 mL microwave vial, a mixture of aromatic azides (1.0 eq.) and substituted aromatic acetylenes (1 eq.) were dissolved in 5 mL (1:1, v/v) of tert-butanol/water with a magnetic stir bar. Aqueous solutions of CuSO4·5H2O (2 mg, 0.0080 mmol, 0.02 eq., in 0.5 mL water) and sodium ascorbate (10 mg, 0.0401 mmol, 0.1 eq., in 1.0 mL of water) were added to the reaction, in that order. The vial was capped properly and placed in the microwave at 200 W, 80–90° C, 18 PSI for 20 min. Upon cooling the vial to room temperature, the resulting mixture was placed in an ice bath and diluted with cold deionized (D. I.) water (~10 mL) to induce precipitation. Vacuum filtration followed by trituration of the residue with D. I. water (10 mL, 2 times) yielded the pure solid product. The reaction was monitored by Thin Layer Chromatography (TLC) and the characterization was carried out by proton (^{1}H), carbon (^{13}C), and fluorine (^{19}F) nuclear magnetic resonance spectroscopy (NMR).

General Scheme of the Triazoles and compounds synthesized:

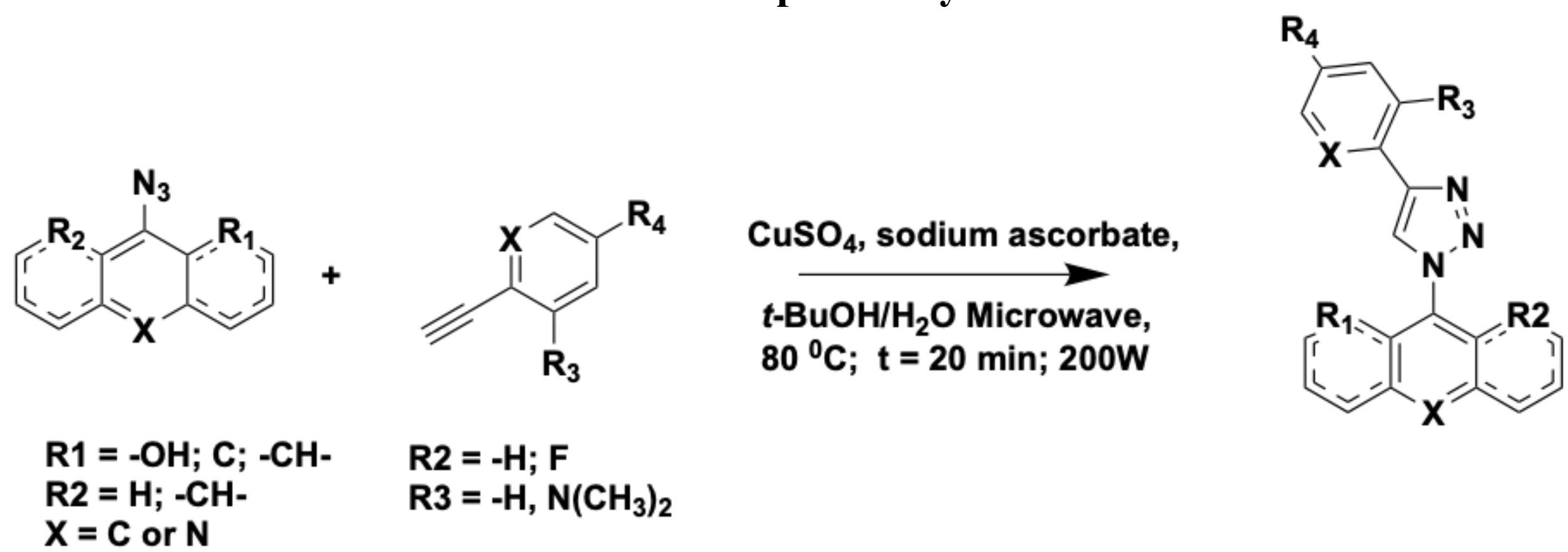

Azide **Alkyne** **Triazoles**

#	Azide	Alkyne
1	X = C; R1 = -OH; R2 = -H	X = N; R3 = H; R4 = H
2	X = C; R1 = -OH; R2 = -H	X = N; R3 = F; R4 = H
3	X = N; R1 = -CH-; R2= -H	X = C; R3 = H; R4 = $N(CH_3)_2$
4	X = N; R1 = -CH-; R2= -CH-	X = C; R3 = H; R4 = $N(CH_3)_2$
5	X = N; R1 = -CH-; R2= -CH-	X = N; R2 = F; R3 = H

Characterization of Triazoles

All chemicals and reactants were obtained from commercial sources and used without further purification.

- **NMR Spectroscopy**
  NMR spectra were recorded on a Agilent MR400DD2 spectrometer, with a multinuclear probe with two RF channels and variable temperature capability (^{1}H-NMR: 400 MHz , ^{13}C-NMR: 100 MHz) and on JEOL ECZL- 400 S NMR with 24 position Auto Sample Changer, 400 MHz FG/ROHFX DIG Autotune Probe (19F-NMR: 376 MHz). The solvent used was CD_3CN or DMSO-d6 (99.8%) with TMS as an internal standard set at 0 *ppm* in^{1}H-NMR, ^{13}C NMR, and ^{19}F-NMR spectra. The NMR signals are reported in parts per million (*ppm*) relative to the residual in the solvent. Signals are described with multiplicity, singlet (*s*), doublet (*d*), triplet (*t*), triplet of doublet (*td*), quartet (*q*) and multiplet (*m*); coupling constants (*J*; *Hz*) and integration.

- **Thin Layer Chromatography**
  All reactions were monitored by thin–layer chromatography (TLC) using Merck silica gel plates 60 F 254; visualization was accomplished with UV light and/or staining with appropriate stains (Iodine, Vanillin).

- **Melting Point**
  Melting points were measured with Vernier Melt Station using Vernier LabQuest 2 and are uncorrected.

- **UV-Vis and Fluorescence Spectroscopy**
  Room temperature absorption and steady state fluorescence measurements are performed using a Shimadzu UV-2450 spectrophotometer and PerkinElmer LS55 with well plate reader fluorimeter respectively. Concentration of triazoles are kept at ~5.0×10^{-5} mol dm^{-3} in solvent to avoid any possible intermolecular effect. The solvents used are of HPLC grade and Millipore water has been used for the experiment.

- **High-Resolution Mass Spectrometry**
  (HRMS) spectra were recorded with the use of the Shimadzu LCMS-9030 quadrupole time-of-flight (Q-TOF) spectrometer (ionization by electrospray, positive ion detection). All the experiments are performed at ambient temperature (27 ^{0}C) with air-equilibrated solutions.

- **Infrared Spectroscopy**
  A Nicolet Smart iTX was used to collect sample information. The raw output was then processed to %transmittance.

- **X-ray Crystallography**
  The single x-ray crystal structure measurements were made on a Bruker D8 Venture Photon 100 diffractometer using Mo-Kα radiation.

Computational Methods

- **SMILES -** Students were introduced to SMILES representations. They used the PubChem Sketcher to draw their molecules and convert them to SMILES. https://pubchem.ncbi.nlm.nih.gov//edit3/index.html

- **Swiss ADME -** After converting their molecules to SMILES, students used the web-based ADME prediction server to determine properties of their triazoles. Students learned how to interpret results from this such as a brief description of druglikeness (e.g., Lipinski rules). https://www.swissadme.ch/

- **Molecular Docking with CBDock2 -** To help the students propose a molecular mechanism of inhibition, they were introduced to basic molecular docking. As most students have different backgrounds and comfort with computational tools, we opted to use a web-based docking server, CBDock2. CBDock2 uses Autodock VINA but simplifies the process by only requiring upload of the receptor and the ligand file. For students working on the protein without an available crystal structure, they were directed to use the AlphaFold Server. Some instruction was provided in lecture to ensure that all students can try these tools if they wanted.
http://183.56.231.194:8001/cb-dock2/index.php
https://alphafoldserver.com/welcome

Protein Biochemistry and Enzymatic Assays

- **Protein Expression -** Recombinant expression of all proteins used in the CURE were expressed using synthetic plasmid constructs purchased from GenScript (Piscataway, NJ) and codon-optimized for use in *E. coli*. Commercially-available strains were used for IPTG-induced expression of the proteins. Students have all taken the first of the biochemistry series at this point and all have experience with routine protocols such as transformation, growth, and expression. Students were given the opportunity to test some conditions, although optimization was not a key component of their project. This includes changing expression temperature, induction optical density, and IPTG concentration. As the optimization requires a lot of time, they worked with current research students in this area. For this section, they used incubator/shakers, centrifuges, and UV-Vis spectrophotometer.

- **IMAC Purification -** Constructs used for protein expression have 6X-His tags enabling purification by immobilized metal affinity chromatography (IMAC). For their projects, students were only required to perform a single-step purification using Ni-NTA. Options were provided for students to perform a reverse IMAC to cut the tag and size exclusion chromatography, as these are also covered in the accompanying BCHM3100 lecture. For this section, students were able to use a sonifier, high-speed centrifuge, and FPLC.

- **Protein Quantification and Purity Analysis** - To follow the purification process, students ran SDS-PAGE on samples collected throughout the purification. Students also used a NanoDrop and UV-Vis spectrophotometer in this section. This also served as an illustration of concepts from the lecture, specifically a single-channel vs. multi-channel instrumentation, where the NanoDrop provides a single number but they were able to collect a wavelength scan using the spectrophotometer.

- **Colorimetry-based Activity Assay** - To evaluate the phosphatase activity of the proteins, two synthetic substrates were readily made available to the students - para-nitrophenylphosphate (pnpp), a colorimetric substrate and 6,8-difluoro-4-methylumbelliferyl phosphate (DiFMUP), a fluorimetric substrate. Students were instructed to try pnpp first as it is cheaper and the assay is easier to perform, and both proteins purified were active against it. Students also verified that both PTPs can be inhibited by a general PTP inhibitor, vanadate. For this section of the lab, students used UV-Vis spectrophotometers and a microplate reader. Enzyme kinetics was also done to determine the Michaelis-Menten constants against pnpp for use in their inhibition assays.

- **Inhibition Assays** - After optimizing their assays, students proceed to test the inhibitory activity of their triazoles against their PTP. This was all done using the microplate reader. Students were required to perform at least two triplicates on different days. A single-dose inhibition assay was first performed at 100 µM final concentration of the compound, followed by a dose-dependence assay from 1 nM to as high as 500 µM as compound solubility would permit.

Additional (Optional) Activities

- **Molecular Dynamics Simulations using GROMACS** - A pair working on one of the proteins without an available experimental structure opted to add an additional computational component to their project, by performing molecular dynamics (MD) simulations on their protein in apo and in complex with their inhibitor. For this, they used CHARMM-GUI to set up the simulations and GROMACS to perform the simulations using the university's computing cluster. Built-in GROMACS tools were used to analyze the trajectories. https://www.charmm-gui.org/

- **Computational structure-activity relationships** - Several of the groups chose to perform computational structure-activity relationships. They generated additional triazoles computationally based on their initial structure and based on initial docking results. They then performed additional docking experiments using CBDock2.

Sample Course Material

Following are sample slides used to introduce students to the lab activities and lab safety.

How to prepare for the lab sessions?

Previous Week (Protocols, Handouts will be provided)	In and Right after the Lab
Plan Experiment in Lab Notebook	Be Ready to Execute Experiment
Prepare Data Tables in Lab Notebook	Collect All Data Properly (Notes!)
Read Manual for Instruments Used	Clean Up + Prepare for Next One
Be Familiar with Expected Outcomes	Analyze All Data and Write Up Lab

Only these three will happen inside the lab. The rest are out of the lab.

Always come in prepared - know the goals for the week, know the expected outcomes, be familiar with potential pitfalls, be familiar with the instrument you will use, notebook should have protocol and data sections ready

Expectations for Lab Safety and Lab Etiquette

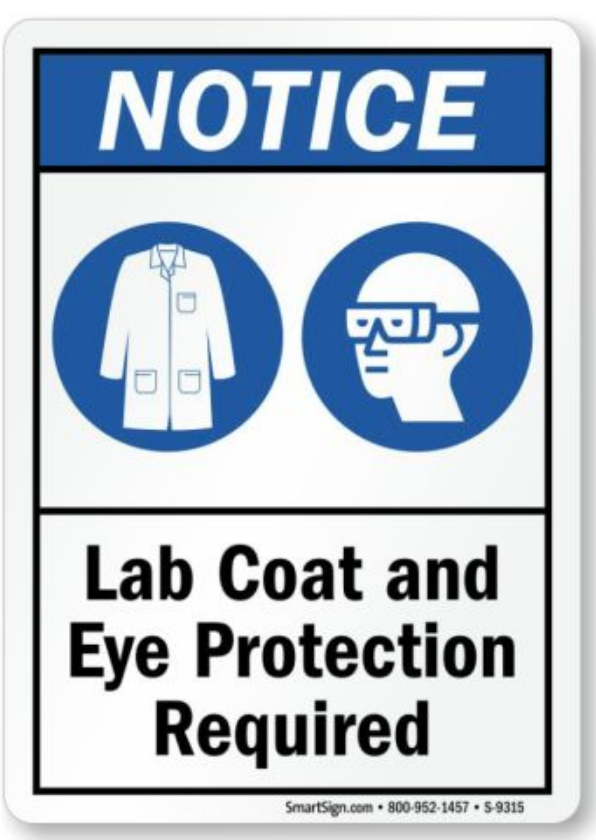

- If you have goggles and lab coat from previous lab or research, you can use those. If not, let us know.
- Part of preparing for lab is knowing the hazards of the chemicals you will be working with. This is expected and required to be part of your lab notebook.
- We will strictly follow the safety guidelines of the Biochemistry, Chemistry, and Physics department.
- *Additional safety precautions will be provided as needed (Dr. Landge).*

Expectations for your Lab Notebook (and example of a good one!)

While the lab is done in pairs, all participants are required to maintain an individual lab notebook. Lab notebooks are critical aspects of research life. *Note: Notebooks will be provided for everyone; you do not need to purchase any.*

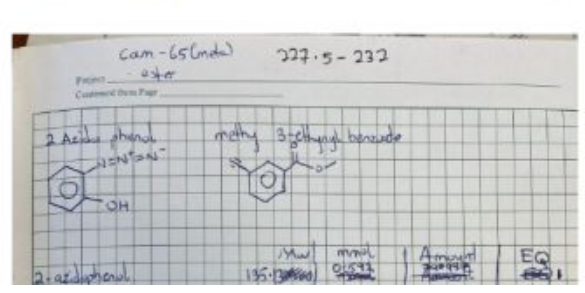

Semester-long Writing Workshop: Improving Scientific Writing

A template for an American Chemical Society (ACS) format manuscript is already available on Folio, Writing Workshop Module.

Topic/Agenda
Whitepaper - Overview of Project (more later)
Outline and Draft of Introduction
ACS-formatted List of References (on-going)
How to Write An Abstract (Drafts Encouraged)
Draft of Abstract (mostly Syn), Title, Introduction
Methods (on-going)
How to Make Figures (at least 2 Figures submitted)
Results and Discussion
Writing-in-Progress Submission 1 of 3
Writing-in-Progress Submission 2 of 3
Writing-in-Progress Submission 3 of 3
Final Paper Deadline

Example of Student Final Poster Presentation

Note that identity of the molecules and protein are redacted as they will be the subject of a future publication with the student scholar.

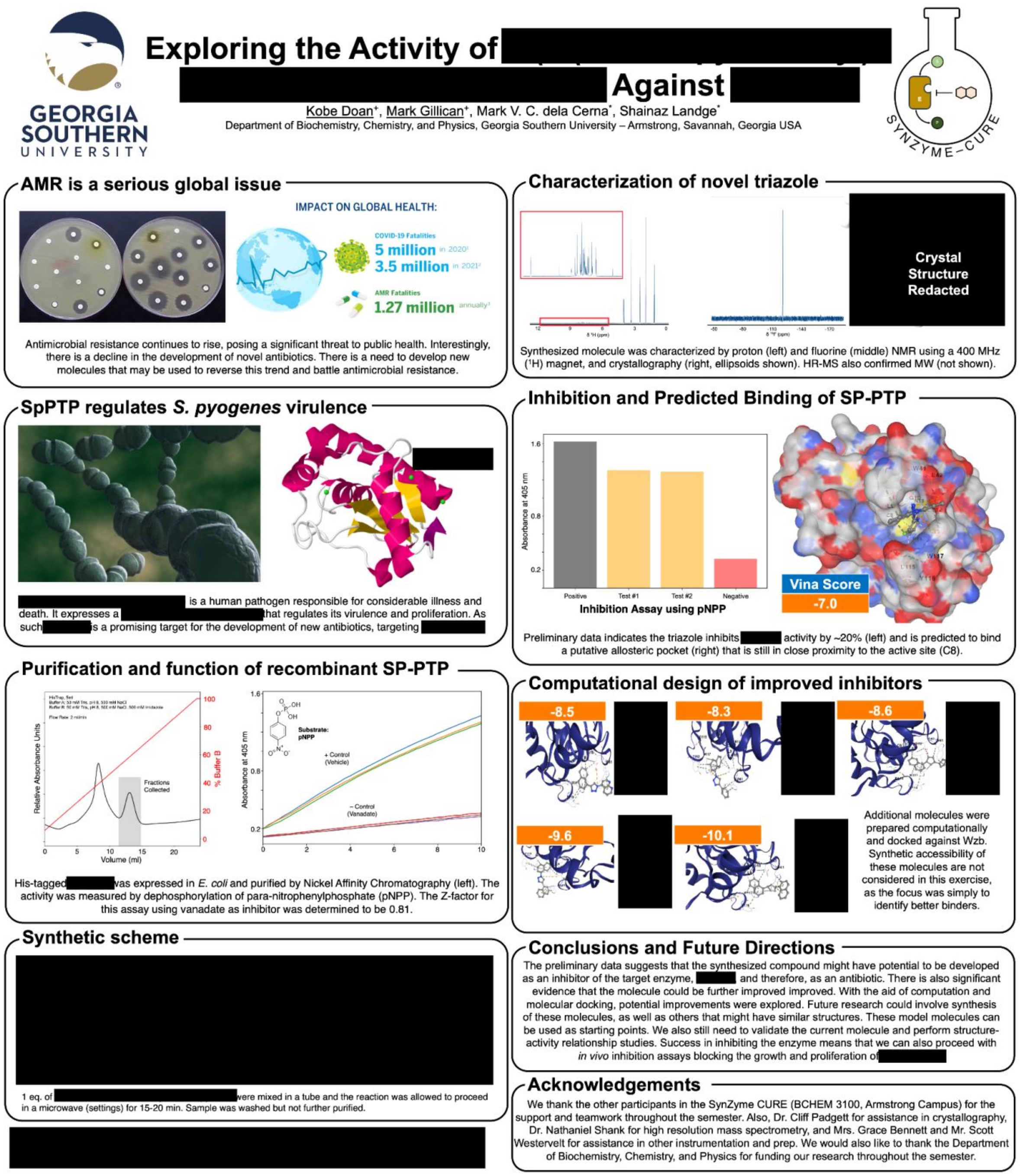

Pocket characterization of [redacted] and its potential inhibition via 1,2,3 triazole derivatives.

Genell Drummonds[1], Oscar Sistos Barron[1], Mark Vincent dela Cerna[1] , Shainaz Landge[2]
[1]Department of Biochemistry, Chemistry, and Physics, Georgia Southern University – Armstrong; [2] Department of Biochemistry, Chemistry, and Physics, Georgia Southern University - Statesboro

At a glance…

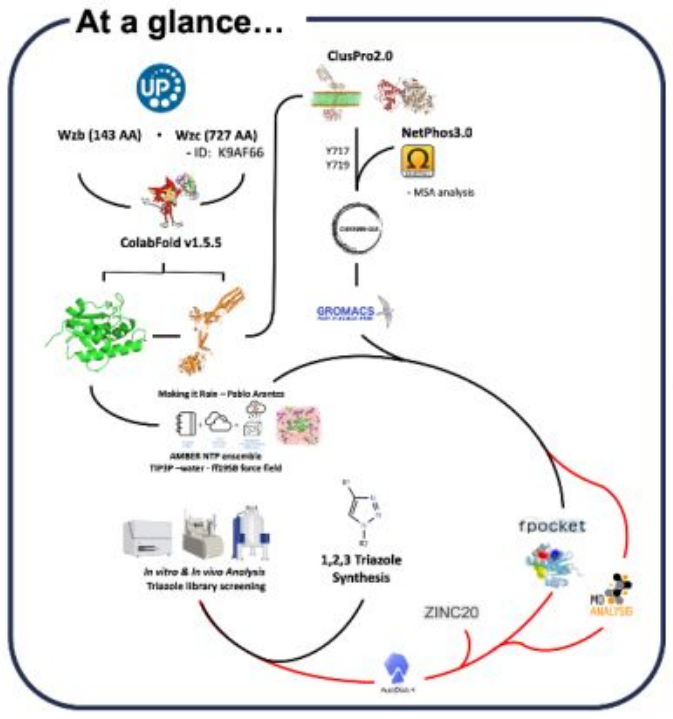

Protein Purification

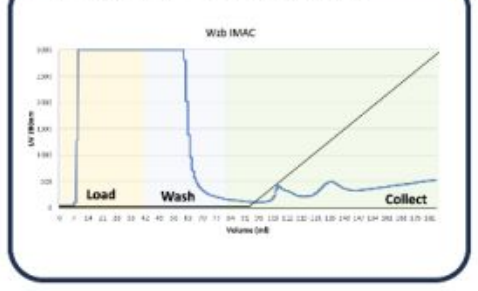

Pocket Identification in Wzb

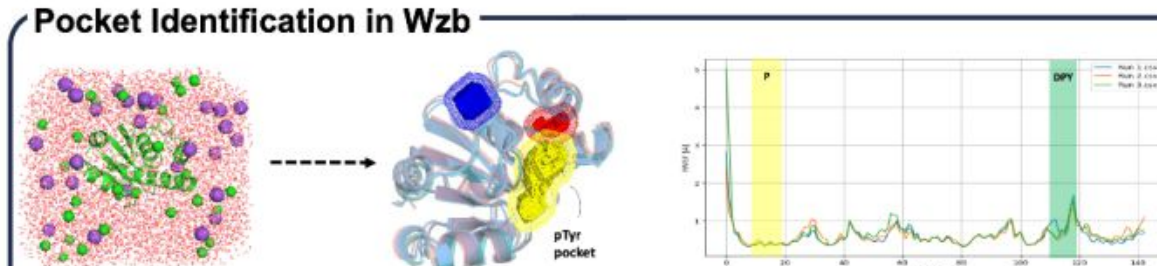

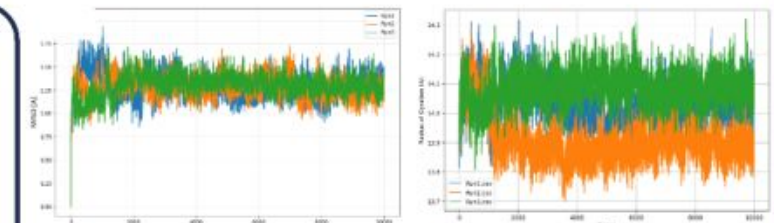

pTyr pocket description

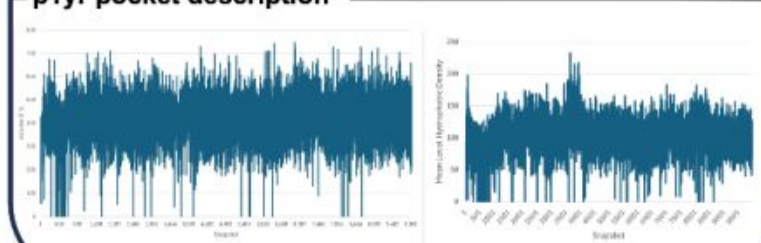

Inhibitory properties of derivatives

[redacted]

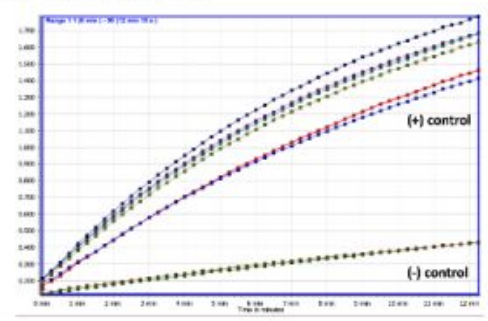

Synthesis of 1,2,3-triazoles

[redacted]

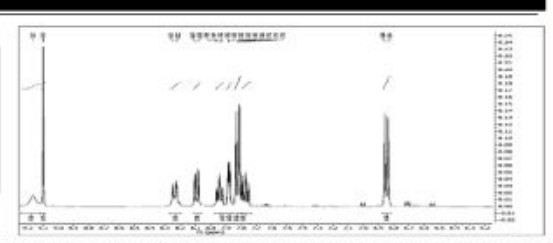

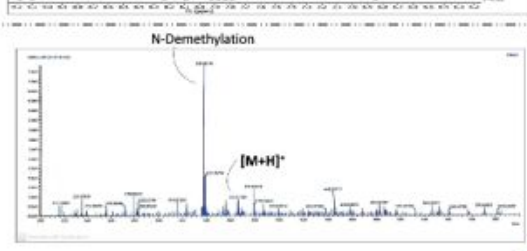

Molecular docking & ADMET analysis

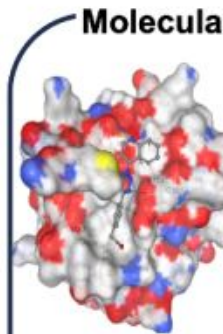

TSJ	Property	OPEA
High	GI Absorption	High
0	Linpinski-Rule violations	0
0	Lead-likeness violations	2
Yes	Structural Alerts (PAINS+brenk)	Yes
Yes	CYP1A2 inhibitor	Yes
Yes	CYP2C9 Inhibitor	Yes
Yes	CYP3D4 Inhibitor	Yes
400	LD50 (mg/kg)	400

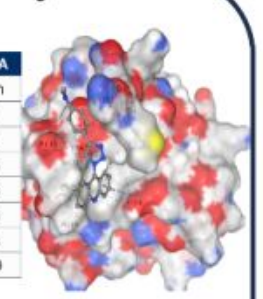

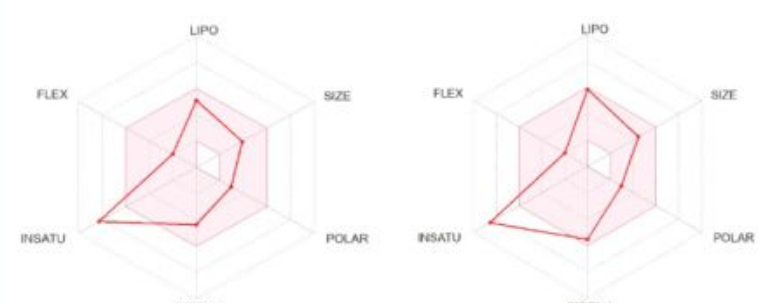

Future Directions

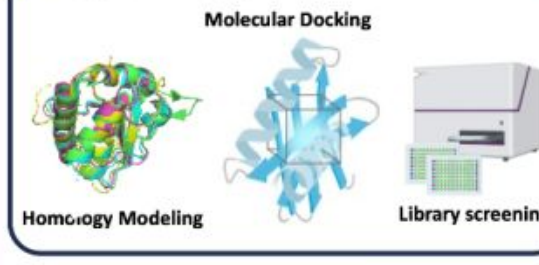

[redacted]

Acknowledgements

We would like to thank the support from the department of Biochemistry, Chemistry, and Physics for the realization and funding of this project. Special thanks for Dr. dela Cerna and Dr. Landge, as well as their respective research groups. Additional thanks to Dr. Quillian, Dr. Padgett, and Dr. Shank for their help in the data characterization of these triazole derivatives.

Table 1: Student Demographics

	SynZyme CURE sample
Sample Size	8
Men	4 (50%)
Women	4 (50%)
American Indian	0 (0%)
Asian	4 (50%)
Black or African American	2 (25%)
Native Hawaiian or other Pacific Islander	0 (0%)
White	3 (37.5%)
Hispanic or Latino/Latina	1 (12.5%)
Junior	2 (25%)
Senior	6 (75%)
Nonparenthetical values represent total responses.<br>Nonparenthetical values represent a percentage of the total sample.	

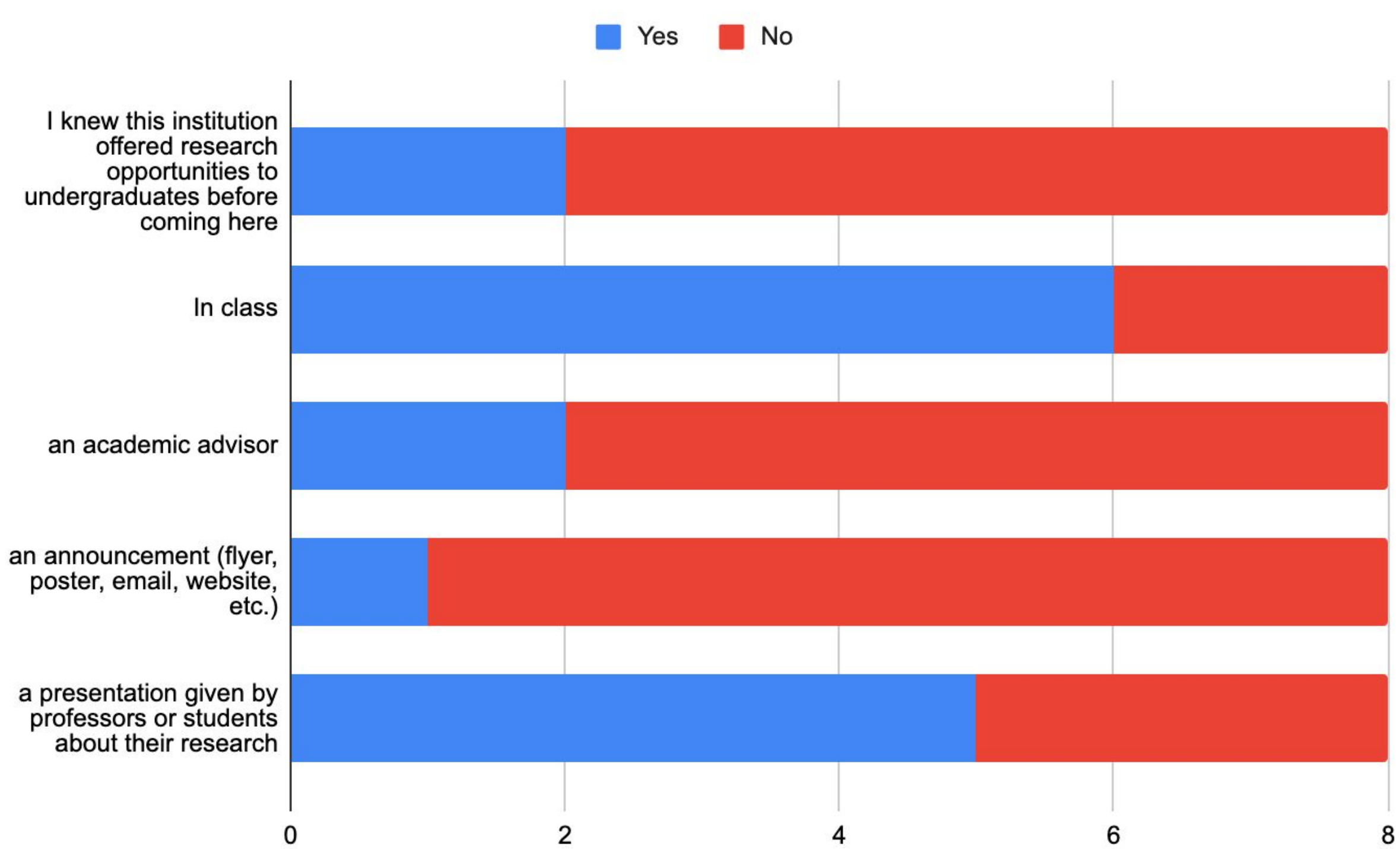

Figure S1: Research Opportunities on Campus

Table 2: Student Commitment to Research

19.1 **Academic Year Research** How many times have you participated in ACADEMIC YEAR research?				
**Never Participated**	**1 semesters**	**2 semesters**	**3 semesters**	**4 or more semester**
**(2)**	**(2)**	**(1)**	**(3)**	**(0)**
**20.1 Summer research** How many times have you participated in Summer research?				
**Never participated**	**1 summer**	**2 summers**	**3 summers**	
**(6)**	**(1)**	**(1)**	**(1)**	
**21.1** **[Academic year = AY; Summer = Sum; Weeks = Wks] Research Experience** For your most recent research experience, how long did you do research?				
**Part of AY**	**All of AY**	**During sum: 5 wks or less**	**Sum: 6-8 wks**	**Sum: 9 wks or more.**
**(4)**	**(1)**	**(1)**	**(0)**	**(2)**
**22.1 Research activities** How many hours per week did you work at research-related activities in your most recent research experience?				
**1-5 hours**	**6-10 hours**	**11-15 hours**	**16 - 20 hours**	**21 or more hours**
**(3)**	**(1)**	**(2)**	**(1)**	**(1)**
23.1 **Hours with mentor** On average, how many hours per week did you spend talking with your most recent research mentor?				
**None**	**1 hour**	**2 hours**	**3 hours**	**4 or more hours**
**(0)**	**(2)**	**(5)**	**(0)**	**(1)**

Survey Assessment of Learning Gains (SALG)- Master Instrument

Undergraduate Research Student Self-Assessment (URSSA) items 1-4

Likert scale 1-5

no gains   a little gain   moderate gain   good gain   great gain

Domains	Item #		Mean
Thinking and Working Like A Scientist	1.1	Analyzing data for patterns.	4.14
	1.2	Figuring out the next step in a research project.	4.37
	1.3	Problem-solving in general.	4.25
	1.4	Formulating a research question that could be answered with data.	4.5
	1.5	Identifying limitations of research methods and designs.	4.5
	1.6	Understanding the theory and concepts guiding my research project.	4.62
	1.7	Understanding the connections among scientific disciplines.	4.5
	1.8	Understanding the relevance of research to my coursework.	4.5
Personal Gains Related to Research	2.1	Confidence in my ability to contribute to science.	4.5
	2.2	Comfort in discussing scientific concepts with others.	4.0
	2.3	Comfort in working collaboratively with others.	4.25
	2.4	Confidence in my ability to do well in future science courses.	4.5
	2.5	Ability to work independently.	4.5
	2.6	Developing patience with the slow pace of research.	4.25
	2.7	Understanding what everyday research work is like.	4.62
	2.8	Taking greater care in conducting procedures in the lab or field.	4.37
Skills	3.1	Writing scientific reports or papers.	4.12
	3.2	Making oral presentations.	3.5
	3.3	Defending an argument when asked questions.	3.62
	3.4	Explaining my project to people outside my field.	4.37
	3.5	Preparing a scientific poster.	4.5
	3.6	Keeping a detailed lab notebook.	4.0
	3.7	Conducting observations in the lab or field.	4.25
	3.8	Using statistics to analyze data.	3.87

	3.9	Calibrating instruments needed for measurement.	4.12
	3.10.	Working with computers.	3.62
	3.11	Understanding journal articles.	4.5
	3.12	Conducting database or internet searches.	4.75
	3.13	Managing my time.	4.0
**none** **a little** **some** **a fair amount** **a great deal**			
	4.1	Engage in real-world science research	4.25
	4.2	Feel like a scientist.	4.25
	4.3	Think creatively about the project.	4.25
	4.4	Try out new ideas or procedures on your own.	4.12
	4.5	Feel responsible for the project.	4.62
	4.6	Work extra hours because you were excited about the research.	3.87
	4.7	Interact with scientists from outside your school.	2.0
**Changes in Attitudes and Behaviors**	4.8	Feel a part of a scientific community.	4.0
**Remaining part of the Adapted Survey Assessment of Learning Gains (SALG)**<br>**Likert scale 1-4**<br>**Poor** **Fair** **Good** **Excellent**			
	5.1	My working relationship with my research mentor	3.5
	5.2	My working relationship with research group members.	3.5
	5.3	The amount of time I spent doing meaningful research.	3.62
	5.4	The amount of time I spent with my research mentor.	3.0
	5.5	The advice my research mentor provided about careers or graduate school.	2.83
	5.6	The research experience overall.	3.5
**Student Research Experience**	5.7	Please comment on any of these aspects.	
**Yes and No Questions**			**(Y/N)**
	6.1	I presented a talk or poster to other students or faculty	8/0
	6.2	I presented a talk or poster at a professional conference	1/7
**Recent Research Experience**	6.3	I attended a conference	4/4

	6.4	I wrote or co-wrote a paper that was published in an academic journal	0/8
	6.5	I wrote or co-wrote a paper that was published in an undergraduate research journal	0/8
	6.6	I will present a talk or poster to other students and faculty	3/5
	6.7	I will present a talk or poster at a professional conference	1/7
	6.8	I will write or co-write a paper to be published in an academic journal.	2/6
	6.9	I will write or co-write a paper to be published in an undergraduate research journal.	2/6
	6.10	I won an award or scholarship based on my research	0/8
**Likert scale 1-4**<br>**Strongly disagree** **Disagree** **Agree** **Strongly agree**			
**Research Experience**	7.1	Doing research confirmed my interest in my field of study.	3.5
	7.2	Doing research clarified for me which field of study I want to pursue.	3.37
	7.3	My research experience has prepared me for advanced coursework or thesis work	3.37
	7.4	My research experience has prepared me for graduate school.	3.37
	7.5	My research experience has prepared me for a job.	3.25
**Likert scale 1-5**<br>**not more likely** ***a little more likely*** **somewhat more likely** ***much more likely*** **extremely more likely**			
**Prior or Recent Research Experience**	8.1	enroll in a Ph.D. program in science, mathematics or engineering?	3.62
	8.2	enroll in a masters program in science, mathematics or engineering?	4.0
	8.3	enroll in a combined M.D/Ph.D program 8.4 enroll in medical or dental school?	3.0
	8.4	enroll in medical or dental school?	2.33
	8.5	enroll in a program to earn a different professional degree (i.e. law, veterinary medicine, etc.)	1.83
	8.6	pursue certification as a teacher?	1.42
	8.7	work in a science lab?	4.0
**Open Ended Questions**			

**Other.** Please state your intended degree and, compared to your intentions before doing research, How likely you are now to enroll in a graduate program leading to an advanced degree.	8.8		
**Future career plans** How did your research experience influence your thinking about future career and graduate school plans? Please explain.	9.1		
**Other gains** Did you make other gains from doing research that we didn't mention? If so, please briefly describe these.	10.1		
**Likert scale 1-4**<br>**Very dissatisfied** **Somewhat dissatisfied** **Somewhat satisfied** **Very satisfied**			
	11.1	Information available to help me choose a research project.	3.87
	11.2	Lab or field equipment.	3.37
	11.3	Support and guidance from program staff.	3.83
	11.4	Support and guidance from my research mentor.	3.87
	11.5	Support and guidance from other research group members.	3.87
**Research Program**	11.6	Research group meetings.	3.62
**Likert scale 1-5**			

Did not do this activity Not at all A little A good amount A great deal			
Training Sessions	12.1	Workshop(s)on science writing and presentation.	3.62
	12.2	Training in library/internet/database search methods.	3.12
	12.3	Safety Training	3.62
**Yes and No Questions**			Y/N
Research opportunities on campus?	15.1	I knew this institution offered research opportunities to undergraduates	2/6
	15.2	In Class	6/2
	15.3	An academic advisor	2/6
	15.4	an announcement (flyer, poster, email, website, etc.)	1/7
	15.5	a presentation given by professors or students about their research	5/3
**Yes and No Questions**			Y/N
Motivation to do Research	16.1	explore my interest in science	7/1
	16.2	gain hands-on experience in research	6/2
	16.3	clarify which field I wanted to study	7/1
	16.4	clarify whether graduate school would be a good choice for me	4/4
	16.5	clarify whether I wanted to pursue a science research career	6/2
	16.6	have a good intellectual challenge	7/1
	16.7	work more closely with a particular faculty member	1/7
	16.8	participate in a program with strong reputation	5/3
	16.9	get good letters of recommendation	4/4
	16.10	enhance my resume	6/2
**Likert scale 1-5 (In SI Table 2)**<br>**Never Participated 1 semesters 2 semesters 3 semesters 4 or more semesters**			
Academic Year Research	19.1	How many times have you participated in ACADEMIC YEAR research?	
**Likert scale 1-4 (In SI Table 2)**			

Never participated 1 summer 2 summers 3 summers			
Summer research	20.1	How many times have you participated in Summer research?	
Likert scale 1-5 [Academic year = AY; Summer = Sum; Weeks = Wks] (In SI Table 2)<br><br>Part of AY All of AY During sum: 5 wks or less Sum: 6-8 wks Sum: 9 wks or more.			
Research Experience	21.1	For your most recent research experience, how long did you do research?	
Likert scale 1-5 (In SI Table 2)<br><br>1-5 hours 6-10 hours 11-15 hours 16 - 20 hours 21 or more hours			
Research activities	22.1	How many hours per week did you work at research-related activities in your most recent research experience?	
Likert scale 1-5 (In SI Table 2)<br><br>None 1 hour 2 hours 3 hours 4 or more hours			
Hours with mentor	23.1	On average, how many hours per week did you spend talking with your most recent research mentor?	

27. Overall Feedback

27.1 What would have made your research experience better? _______

27.2 What would improve the undergraduate research program, overall? ______

Demographics **(In SI Table 1)**

28. Gender	Male	Female	Do not want to report
28.1 What is your gender?			
29. What is your race? (choose all that apply)	yes	no	Do not want to report
29.1 American Indian			
29.2 Asian			
29.3 Black or African American			
29.4 Native Hawaiian or other Pacific Islander			
29.5 White			
29.6 Other (please specify)	________________________		
30. What is your ethnicity?	yes	no	Do not want to report
30.1 Hispanic or Latino/Latina			

**Box 1. Responses to Open-Ended Questions Pertaining to Post-Graduation Plans and Additional Gains**
Question 1: *What is your intended degree? Compared to your intentions before doing research (CURE), how likely are you to enroll in a graduate program leading to an advanced degree.*
“Biochemistry, much more likely.”
“I was always interested in applying to a graduate program but this research project confirmed my interest and assured me that it would be a good fit for me and my goals.”
“I plan to graduate with a Bachelor's degree in Biochemistry, and although I had previously planned to enroll in a graduate program, this class helped as good practice to be more confident in my decision.”
“I am currently pursuing an undergraduate degree in Biochemistry, and my intended path after graduation is to continue toward an advanced degree in a research-focused field. Before participating in this research experience, I was somewhat uncertain about whether graduate school was the right next step for me. I knew it was an option, but I wasn’t fully confident in my readiness. After completing this research experience, I am now much more likely to enroll in a graduate program. The hands-on work, problem-solving, and exposure to real research environments helped me realize that I truly enjoy scientific investigation. This experience strengthened my motivation to continue my education and pursue advanced training that will allow me to grow as a researcher in biochemistry.”
“Chemistry, not sure at this point”
“My intended degree before and after doing research is in biochemistry. Enrollment in a graduate program for me is highly likely as I would love to continue down the path I am on now.”
Question 2: *Reflect on how their research experience influenced their own thinking about future career and graduate school plans.*
“My research made me interested in pursuing more research before graduating”
“Working on a project from start to finish, utilizing different instruments, and being largely independent during this research experience helped me realize that I definitely want to continue doing research like this and influenced the kind of programs I am planning to apply for. Like drug design.”
“I know that I am not fit for lab work as it is not what I intend to do as something for fun.”
“It has strengthened my willingness to apply to graduate programs as I have enjoyed the overall experience in taking part of a research project.”
“It helped me recognize that research is something I want to continue doing long-term. This experience made graduate school feel more appealing. I gained confidence in my ability to work independently, communicate with team members, and understand advanced laboratory techniques- skills that are essential for success in a Master’s or Ph.D. program. Seeing how research contributes to scientific progress and opens doors to careers in academia, industry, and government further reinforced my interest in pursuing an advanced degree.”
“I am more likely to go to Graduate School, but I will probably not for personal reasons. I have considered going into research a great deal more as a result of this experience.”
“Yes it has, when I started this degree I had intended on using it as a stepping stone for dentistry but after engaging in research, working in a lab is a more enticing and rewarding career path for me.”
Question 3: *Are there additional gains from the CURE not addressed in previous questions?*
“I think I gained the ability to work more collaboratively and set firm time management goals to make sure my work is complete on time.”
“I was able to talk to more science related people and exchange thoughts.”
“Yes. One major gain was increased confidence in my ability to problem-solve and adapt when experiment didn’t go as planned. I also gained a better understanding of how to manage long-term projects, balance multiple responsibilities, and stay organized.”

**Box 2. Additional Student Feedback**
What would have made your research experience better
"Maybe a little more guidance for product characterization but I understand it was a challenge due to it being spread across both campuses".
"I'm not sure if there's anything that could have made the experience better. I already find it as an incredible learning experience."
What would improve the UG research program overall
"More paid positions, to motivate students".
"The undergraduate research program is already strong. The only hiccup was not having access to instruments (particularly the plate reader) readily and I don't think that's an issue to the program itself."

References:

Landge, S.; Philp, J.; Ugboya, A.; Graves, I.; Fasusi, E.; Jordan, K.; Aiken, K.; Sittaramane, V. Evaluation of Ortho-Substituted Bis-Functionalized Triazoles as Tyrosinase Inhibitors: Modulating Dopamine Synthesis and Behavior in Zebrafish. *Med. Chem. Res.* **2024**, *33* (4), 651–662. https://doi.org/10.1007/s00044-024-03209-z.
